# Noise-resistant phase imaging with intensity correlation

**DOI:** 10.1126/sciadv.adh5396

**Published:** 2023-09-22

**Authors:** Jerzy Szuniewicz, Stanisław Kurdziałek, Sanjukta Kundu, Wojciech Zwolinski, Radosław Chrapkiewicz, Mayukh Lahiri, Radek Lapkiewicz

**Affiliations:** ^1^ Institute of Experimental Physics, Faculty of Physics, University of Warsaw, ul. Pasteura 5, 02-093 Warszawa, Poland.; ^2^ CNC Program, Stanford University, Palo Alto, CA 94304, USA.; ^3^ Department of Physics, Oklahoma State University, Stillwater, OK 74078, USA.

## Abstract

Interferometric methods form the basis of highly sensitive measurement techniques from astronomy to bioimaging. Interferometry typically requires high stability between the measured and reference beams. The presence of rapid phase fluctuations washes out interference fringes, making phase profile recovery impossible. This challenge can be addressed by shortening the measurement time. However, such an approach reduces photon-counting rates, precluding applications in low-intensity imaging. We introduce a phase imaging technique which is immune to time-dependent phase fluctuations. Our technique, relying on intensity correlation instead of direct intensity measurements, allows one to obtain high interference visibility for arbitrarily long acquisition times. We prove the optimality of our method using the Cramér-Rao bound in the extreme case when no more than two photons are detected within the time window of phase stability. Our technique will broaden prospects in phase measurements, including emerging applications such as in infrared and x-ray imaging and quantum and matter-wave interferometry.

## Teaser

Spatial intensity correlations are used to perform interferometric phase imaging in the absence of intensity interference fringes.

## INTRODUCTION

Quantitative phase imaging (*1*–*3*) is one of the most effective ways to acquire high-contrast images of transparent objects that absorb or scatter light negligibly. Because optically thin specimens are often encountered in biological and medical imaging, quantitative phase imaging has found immense applications in biological and medical sciences (*4*), such as in cell biology (*5*), immunology (*6*), neuroscience (*7*), and cancer diagnosis (*8*) and prognosis (*9*). Interferometry (*10*) is central to quantitative phase imaging and has been applied to various imaging modalities such as diffraction phase microscopy (*11*), sample-safe defectoscopy (*12*), wavefront sensing (*13*), spatial light interference microscopy (*14*), and three-dimensional (3D) tomography (*15*). Moreover, interferometry is the basis of optical coherence tomography (*16*, *17*) and shearing interferometry (*18*–*20*). For an interferometric measurement, a wave field that has interacted with an object is superposed with a reference field, and the resulting interference pattern is detected by a camera. If the object and the reference fields are mutually coherent, the time-averaged intensity on the camera is given by (*3*, *21*)

$$I(x,y)=I_{r}+I_{o}+2\sqrt{I_{r}I_{o}}\cos[\text{Θ}+\phi (x,y)]$$ (1)

where *I*_r_ and *I*_o_ are intensities of the reference and the object fields, respectively, Θ is the spatially uniform phase that can be changed by introducing a delay between the two fields, and ϕ(*x*, *y*) is the phase profile of the object. Standard interferometric phase imaging techniques are based on the signature of ϕ(*x*, *y*) left in the detected intensity pattern. However, for any such method to be applicable, the object and the reference fields need to be mutually coherent, that is, phase Θ must be stable in time. The method is therefore vulnerable to time-dependent, uncontrollable phase fluctuations. When the phase fluctuates much faster compared to the detection time, the coherence between the object and image fields is effectively lost, and no interference is observed, i.e.

$$I(x,y)=I_{r}+I_{o}$$ (2)

Because there is no information about ϕ(*x*, *y*) in this intensity pattern, the standard phase imaging schemes become inapplicable. One way to avoid the effect of time-dependent phase fluctuations is to shorten the duration of the measurement (*22*). A short measurement time, however, reduces the amount of detected light and is therefore impractical in many cases including, for instance, imaging of photosensitive biological specimens, which require low-intensity light. Furthermore, for interferometric fluorescence superresolution microscopy (*23*), very low-intensity light (*24*) often needs to be superposed. In such cases, any time-dependent phase fluctuations must be avoided because of the relatively long detection time requirement.

Here, we introduce, phase imaging with intensity correlation (PI2C), a quantitative phase imaging technique that is fully resistant to time-dependent phase fluctuations as long as it is possible to detect at least two photons within a time window in which the phase Θ is stable (phase stability time). The advantage of our method stems from the fact that it relies on the intensity correlation rather than intensity measurement, which makes it fundamentally different from standard phase imaging techniques (*25*). Numerous state-of-the-art approaches to low-light imaging (*26*–*30*) rely on quantum light sources. It is crucial that our technique works for easily accessible and robust classical light sources, which markedly broadens its applicability.

**Fig. 1.**
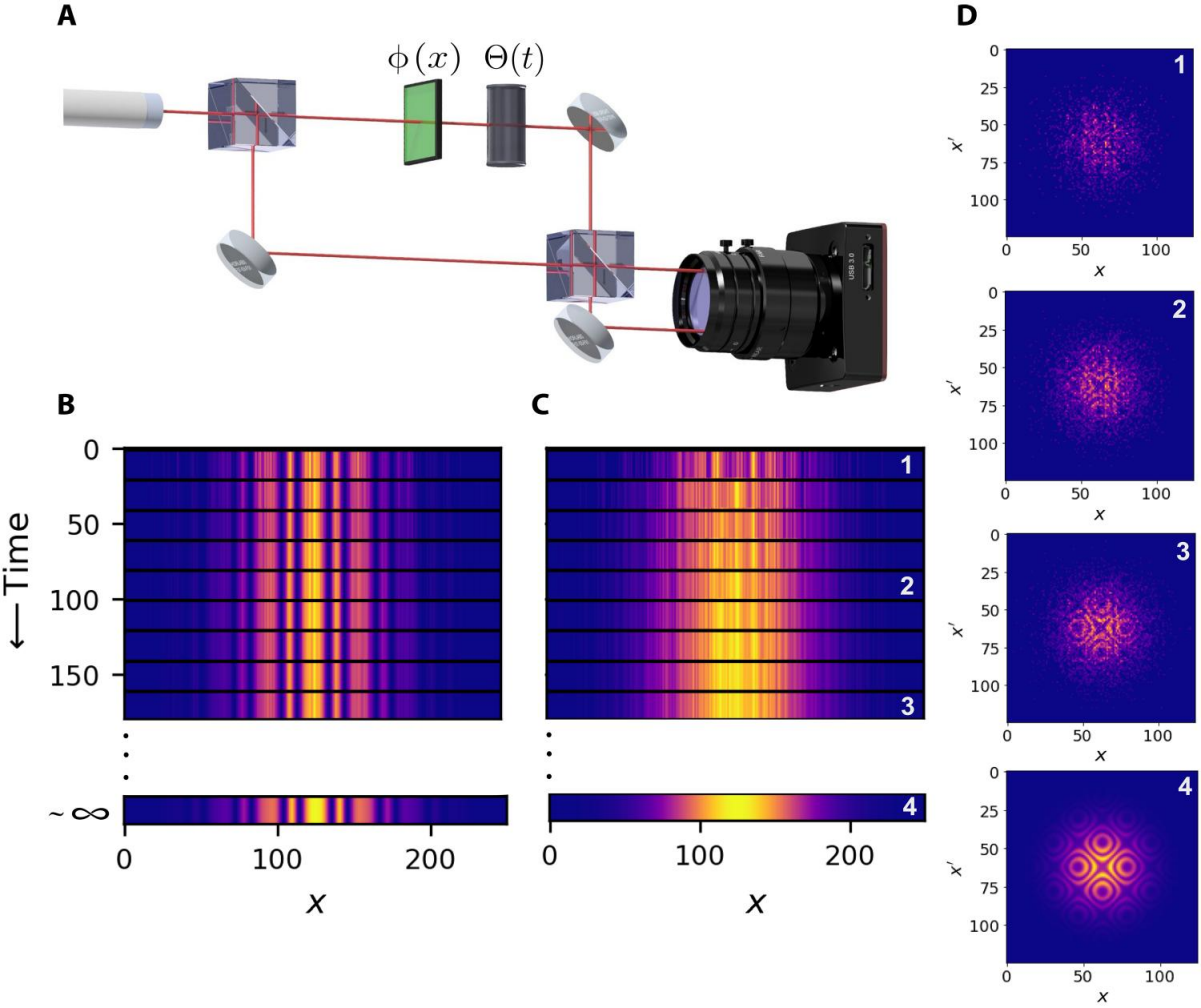
The concept of noise-resistant phase imaging and a simulation of intensity interference fringes compared to intensity correlation fringes. (**A**) Simplified schematic of the experiment. We divide input light into two paths, an object path and a reference path. In the object path, we introduce a spatially varying 1D phase object ϕ(*x*) that we want to image. A time-fluctuating interferometric phase Θ(*t*) can be introduced to the system. For nonfluctuating phase Θ, we can measure high-visibility interference fringes (**B**) (see Eq. 1). For rapidly fluctuating Θ(*t*), we observe the loss of interference visibility with increasing measurement time, whereas for a short measurement time, the signal-to-noise ratio (SNR) is very low because of an insufficient number of detected photons (**C**). In contrast, in our method, the visibility remains constant for an arbitrary acquisition time, which allows us to create high-SNR interferograms (**D**). Images (B) and (C) depict normalized one-photon interference fringes for nonfluctuating and rapidly fluctuating phase scenarios, respectively. Second-order correlation interferograms shown in (D) are created using the same photon detections as standard interferograms presented in (C). Even for rapidly fluctuating phase, where we record only a few photons within the stability time of Θ(*t*), we can retrieve second-order correlation interferograms with high visibility and high signal-to-noise ratio (SNR) that contain full information about the phase profile ϕ(*x*).

The scheme of our experiment is illustrated in Fig. 1A. The object field is superposed with a reference field, and the resulting interference pattern is detected by a camera. A time-dependent phase fluctuation Θ(*t*) is introduced to the reference field. Under these circumstances, no information on ϕ(*x*, *y*) can be retrieved from the intensity pattern given by Eq. 2, and therefore, standard phase imaging techniques become inapplicable. Our phase imaging method is resistant to time-dependent phase fluctuations, provided that the fluctuating phase is spatially invariant throughout the entire sample (*31*). This approach is inspired by higher-order correlation effects observed in interference of light from independent sources (*32*). It relies on measuring intensity correlations of light like in the intensity interferometry technique introduced by Hanbury Brown and Twiss (HBT) (*33*). The HBT method and its generalizations were applied to a variety of light sources (*34*–*42*), and similarly, our technique might be applied in various scenarios including, for instance, laser and thermal light.

We determine the correlation function between the intensities measured at a pair of points (*x*, *y*) and (*x*′, *y*′) (see section S1 for detail)

$$\left\langle \tilde{I}(x,y;t)\tilde{I}(x^{\prime },y^{\prime };t)\rangle \propto 1\pm \frac{1}{2}\cos[\phi (x,y)-\phi (x^{\prime },y^{\prime })\right]$$ (3)

where $\tilde{I}(x,y;t)$ is the instantaneous intensity measured at a point (*x*, *y*) at time *t*. On the right-hand side of Eq. 3, the plus (+) and minus (−) signs apply when the two points of measurement are in the same and different beam splitter outputs, respectively; we also assume that *I*_r_ = *I*_o_ for simplicity. Note that the information about the phase map of the object, which was lost in the intensity pattern (Eq. 2 and Fig. 1C), reappears in the intensity correlation map (Eq. 3 and Fig. 1D).

The second-order intensity correlation map contains the full information required to optimally reconstruct ϕ(*x*, *y*) in the extreme case when only two photons are detected during the phase stability time. Our strategy of reconstructing the actual phase distribution is optimal in this scenario, which we prove rigorously using estimation theory tools, namely, Fisher information and Cramér-Rao (C-R) bound (see sections S1 and S2 for details) (*43*).

## RESULTS

In our experiment, each frame contains on average of ∼15 photons at both outputs of the interferometer per frame. We remove temporal correlations between subsequent frames by randomly permuting the order of frames before further processing; this does not change the performance of our method but allows us to simulate the conditions, in which the phase Θ(*t*) is independently sampled for each consecutive frame. Such conditions occur, when the noise frequency is larger than the delay between subsequent exposures. It is then impossible to retrieve phases using standard interferometric methods. Averaging recorded intensities over multiple subsequent frames would result in a loss of the visibility of the interference fringes. In contrast, we average correlations of detected photons’ positions without any loss of the phase information. Such averaging over multiple frames results in the reproduction of the correlation function (Eq.3), from which we can retrieve the phase profile using standard digital holography methods: Fourier off-axis holography (*44*) has been used in our case. This analyzing mechanism is the essence of our noise-resistant phase imaging technique (see section S4 for details).

**Fig. 2.**
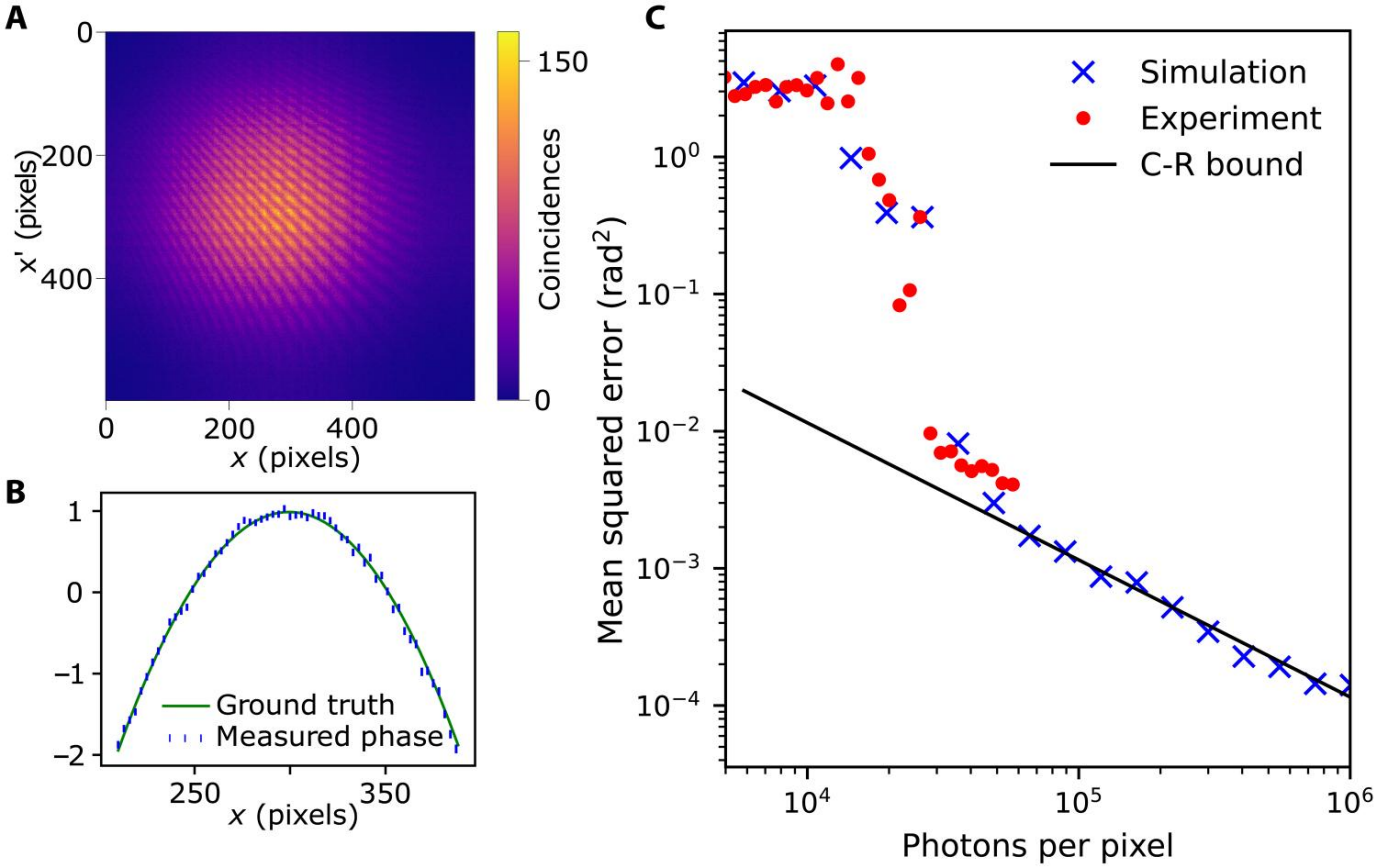
Quantitative analysis of 1D quadratic phase profile reconstruction. (**A**) The experimentally measured coincidence map for a 1D quadratic phase profile, plotted with a solid line in (**B**). The phase reconstructed from experimental data with error bars is also shown in (B). The visibility of the fringes in the correlation map (A) is equal to *v*^2^/2, where *v* = 0.6 (theoretical maximum with classical light is *v* = 1, which leads to 1/2 visibility of the second-order interference). The total number of coincidences detected in the experiment is ∼10^7^. By randomly removing a part of the collected signal, we can check how the MSE, associated with the phase reconstruction, scales with the mean number of photons detected per pixel during the whole experiment (**C**). The MSE from the experiment is then compared with the MSE obtained using a simulated hologram, with the same parameters as in the experiment. We calculate the fundamental C-R lower bound on the MSE, assuming the visibility of hologram fringes to be equal to 0. 6^2^/2 (as in our experiment). When no noise apart from shot noise is present (as in simulation), our method allows the saturation of this fundamental limit for a large enough (∼5 × 10^4^) number of photons detected per pixel. Other possible noise sources, e.g., camera dark counts, may slightly affect the MSE obtained experimentally.

To quantitatively assess the precision of our phase reconstruction method, we measure a 1D quadratic phase object and compare the mean squared error (MSE) obtained in the experiment and in a simulation with the fundamental lower bound for the MSE given by the inverse of Fisher information, namely, C-R bound (see section S2 for details). The measured coincidence map (Fig. 2A) consists of approximately 10^7^ registered photon pairs. To simulate the most extreme case, we use only two randomly chosen photons from each frame for further processing. We estimate the phase profile shape using the collected data and compute the associated MSE, which is the average square distance between the real (ground truth) and measured phase (Fig. 2B). As we show in Fig. 2C, the MSE drops down with the total number of measured photons and eventually reaches the theoretical minimum. This proves that our method of phase estimation is optimal when at most two photons are measured during the phase stability time; notice, that this is the most extreme limit in which one can gain any information about the phase profile.

**Fig. 3.**
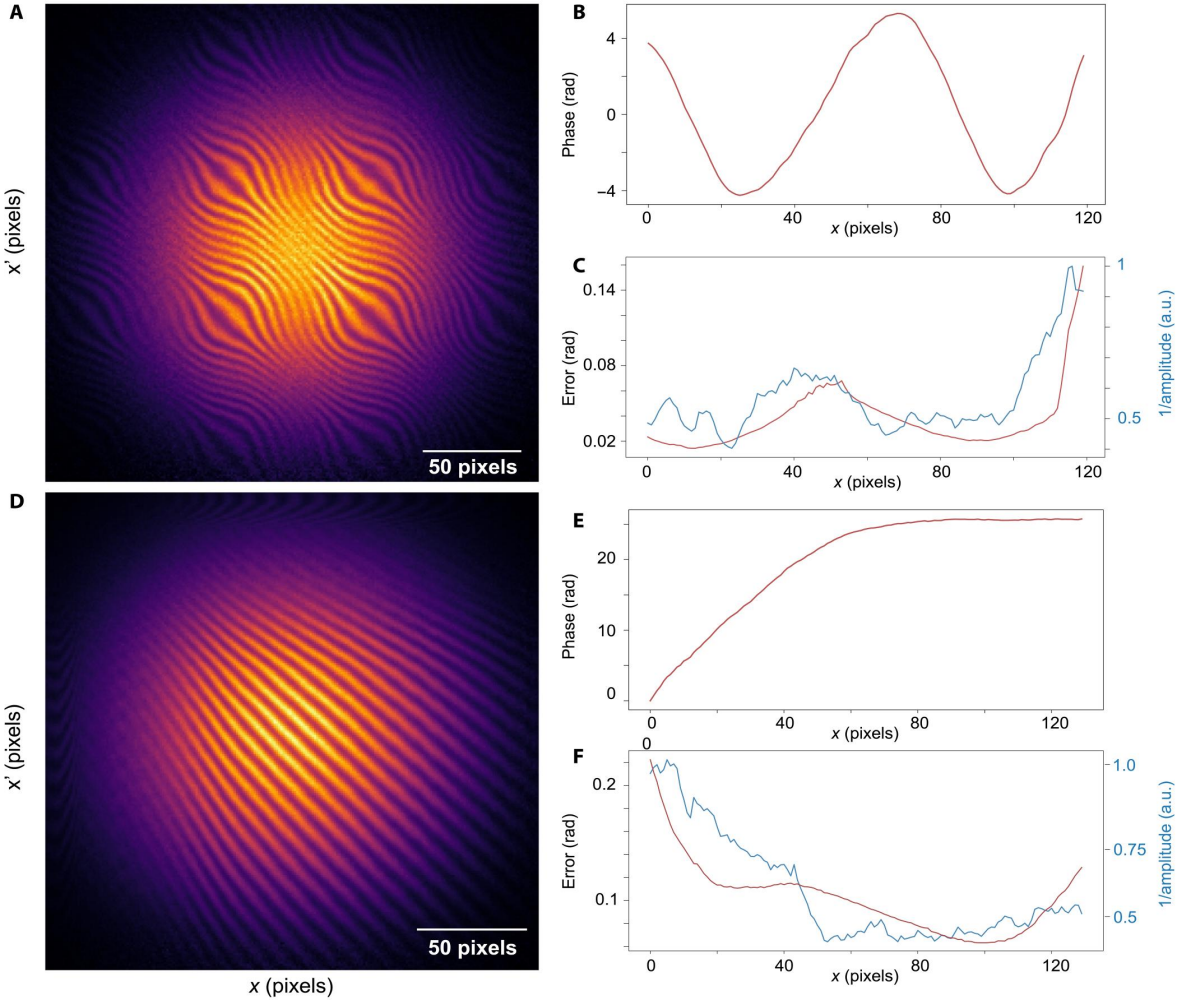
Examples of experimental reconstruction of 1D spatial phase profiles. Sinusoidal and exponential phase profiles are introduced by an SLM. Measured coincidence maps (correlation functions) between outputs of the interferometer for sinusoidal and exponential phases are shown in (**A**) and (**D**), respectively. (**B** and **E**) The aforementioned reconstructed phases. (**C** and **F**) Errors and inverse square root of the intensities. a.u., arbitrary units.

To show the versatility of our method, we now reconstruct spatial light modulator (SLM)–encoded 1D phase profiles ϕ(*x*) of two distinct forms: sinusoidal and exponential. Panels A and D, B and E, and C and F, Fig. 3 represent the measured hologram, the retrieved phase, and the error per pixel for the sinusoidal/exponential phase profile. Systematic errors of phase recovery in both cases are caused by the SLM imperfections.

**Fig. 4.**
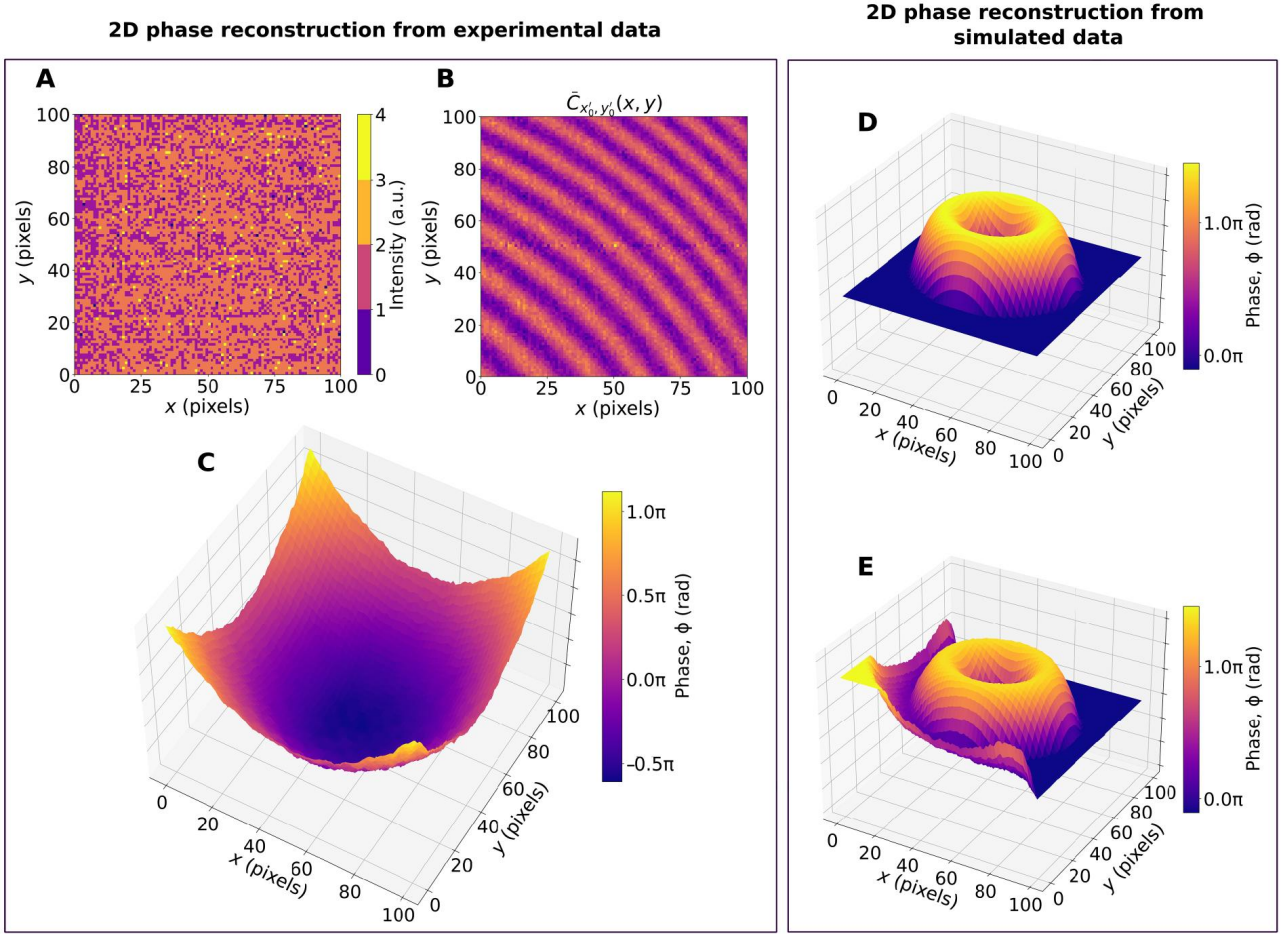
2D phase imaging using intensity correlations. Left: Experimental quadratic phase measurement: (**A**) Fringes are not visible in a single frame image, and frames are dominated by the camera readout noise. (**B**) A slice through the correlation map (at positions *x*′ = 50 pixels, *y*′ = 50) revealing interferometric fringes. (**C**) An average phase retrieved from the complete 4D correlation map. The phase retrieval error is discussed in detail in section S5. Right: 2D phase reconstruction from simulated data. (**D**) 2D phase object (ground truth). (**E**) Retrieved phase profile. More details about the simulation can be found in section S5.

Our method can be readily applied to 2D spatial phase profiles by generating higher-dimensional correlation maps. We demonstrate this by experimentally reconstructing a 2D quadratic phase profile, which is introduced by an optical lens. We detect the photons with a standard complementary metal-oxide semiconductor (CMOS) camera to demonstrate that our method works even in the presence of high noise. Figure 4A presents a randomly selected frame from the intensity measurement, where no interference fringes are visible because of low signal and high noise levels. Figure 4B shows one cross section through the second-order intensity correlation map created from all measured frames; interference fringes are now clearly visible. Figure 4C presents the experimentally retrieved 2D phase map that matches the quadratic phase introduced by the lens with high accuracy. A detailed analysis of the 2D phase retrieval is given in section S5 (*45*). In Fig. 4 (D and E), we also show results of a phase reconstruction from simulated noisy data corresponding to 2D phase imaging of a toroidal phase profile, with Fig. 4D representing the introduced phase while Fig. 4E representing the retrieved phase profile (see section S5.4 for detailed analysis).

## DISCUSSION

We have demonstrated a complete retrieval of phase patterns in the presence of time-dependent high-frequency phase fluctuations using spatial intensity correlation measurement. Because this time-dependent phase fluctuates much faster compared to the detection time, the reference and the object fields can be considered mutually incoherent (*46*). Consequently, our method is applicable to the case when reference and object fields are generated from independent sources (*32*, *38*, *47*, *48*). Although we performed the experiment with laser light, our method is applicable to optical fields characterized by different statistics, such as thermal light. What is more, our technique provides a quantitative phase image and an amplitude image simultaneously. The latter can be easily accessed by summing the intensity correlation interferogram over one of the dimensions. We stress that the presented method optimality is proven using the C-R bound (*43*); all the spatial phase information contained in the detected photons is retrieved with our technique.

High temporal resolution (short exposure time *T*_exp_) is necessary for overcoming the problem of the rapidly fluctuating temporal phases. In our experiment, we used an intensified scientific CMOS (I-sCMOS) camera, which allows us to achieve *T*_exp_ ∼ 20 ns, corresponding to the 50-MHz maximal phase noise frequency that we can deal with. However, our method is not limited to this type of camera and can be implemented using various high-temporal resolution detection platforms. Because of high quantum efficiency, high temporal resolution, and low noise level in recent single-photon avalanche diode (SPAD) array technology (*49*), our method can also be implemented by SPAD arrays.

The fact that phase imaging is possible even in the case of only two photons detected per phase stability time is notable, but we stress that our technique works both in the photon counting regime and by using less accurate intensity measurements. As demonstrated in Fig. 4, one can implement intensity correlation–based phase imaging using an inexpensive CMOS camera.

Our method can be implemented with different degrees of freedom, such as temporal or spectral. The creation of joint probability maps for photon detection times or their detected wavelengths will enable retrieving temporal or spectral phases correspondingly. Such a generalization can find applications in optical coherence tomography. It is also possible to incorporate an additional degree of freedom to a measurement, measuring, for instance, joint temporal-spatial correlation maps.

Equation 3 is only exact when all the values of Θ have the same probability of appearing during the time interval in which the whole measurement is performed. To satisfy this condition for arbitrary temporal phase noise, it is enough to add random uniformly distributed signal oscillating between 0 and 2π to the unknown phase fluctuations Θ(*t*).

Our method, PI2C, opens up possible applications in quantitative phase imaging under low light conditions for microscopy and fundamental research. Unbalanced interferometers, such as the ones used in the time bin encoding, could be of particular interest, as our method enables the use of additional degrees of freedom (for multidimensional information encoding) while filtering out phase fluctuations arising from unmatched optical paths.

Moreover, x-rays and matter waves, because of their short wavelengths, require a very precise alignment and stability of the experimental parameters to interfere (*50*–*53*). For this reason, our phase noise–resistant technique holds great potential for phase-sensitive imaging using x-ray and matter waves (*54*–*56*).

Note that the key ideas and preliminary results of this paper were presented in 2019 at the Rochester Conference on Coherence and Quantum Optics (*31*). While preparing the manuscript, we came across a very recent work that also uses intensity-intensity correlations for phase imaging (*57*).

## METHODS

**Fig. 5.**
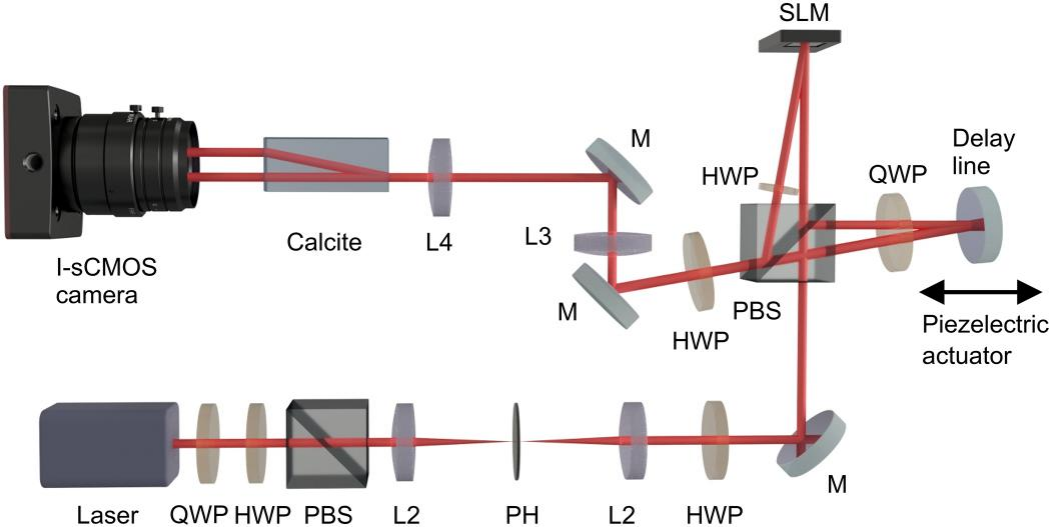
Experimental setup for noise-resistant phase imaging is constructed using polarization-based Michelson interferometer equipped with a 4f imaging system. Light from a 780-nm laser passes through a half-wave plate (HWP or λ/2), quarter-wave plate (QWP or λ/4), polarization beam splitter (PBS) followed by additional λ/2; in this way, we control the beam intensity and polarization. Subsequently, the beam enters the interferometer. In one of its arms, the spatial phase ϕ(*x*) is imprinted onto the beam with the SLM. The mirror in the other adds temporal fluctuations Θ(*t*) caused by a piezoelectric actuator. The beams from both arms are combined at the PBS and pass through a 4f imaging system consisting of two lenses (L3 and L4), which are positioned such that the sample plane is imaged on the camera plane. The calcite polarizer combined with the output HWP (λ/2) acts as a 50/50 beam splitter. The I-sCMOS camera records single photons coming from both outputs of the interferometer.

The idea demonstrated in Fig. 1 is implemented using a polarization-based Michelson interferometer equipped with an imaging system, shown in Fig. 5. We perform experiments with four different phase masks ϕ(*x*) applied to the object beam. We imprint a 1D quadratic phase profile (Fig. 2, A and B) to the beam by placing a cylindrical lens of a focal length *f* = 1000 mm and a 2D quadratic phase profile (Fig. 4, A to C) by placing a spherical lens of focal length *f* = 200 mm in proximity to the mirror instead of the SLM in a slightly modified setup. In two other cases, the sample is realized using the SLM, as it can display an arbitrary phase profile. The SLM imprints 1D sinusoidal (Fig. 3, A to C) and exponential (Fig. 3, D to F) phases to our object beam (see section S3 for details). A time-dependent fluctuating phase Θ(*t*) is introduced in the reference arm to make the object and reference beams effectively incoherent. To register a very low photon flux and to minimize the exposure time, we use an I-sCMOS camera in the case of 1D phase reconstruction. In the case of 2D phase imaging (Fig. 4), we use a standard CMOS camera instead. To get a high interference visibility within a single frame, we keep the camera exposure time (*T*_exp_) low, such that fluctuations of Θ(*t*) are negligible within *T*_exp_. For *T*_exp _∼ 20 ns, this leads to a very low signal-to-noise ratio (SNR) for a single frame, as it contains only a few detected photons on average. However, this does not limit our method because we can sum many correlation maps created from different frames, although the value of Θ(*t*) is different for each frame. As a consequence, we can perform quantitative phase imaging in the presence of temporal fluctuations of frequency of the order ∼1/*T*_exp_ ≃ 50 MHz. This would be impossible for intensity-based interferometry; we would lose either visibility or SNR.

## Supplementary Materials

### This PDF file includes:

Sections S1 to S5 Figs. S1 to S13
