# Supplementary material for: Noise-resistant phase imaging with intensity correlation
Source: Sci Adv. 2023 Sep 22;9(38):eadh5396. doi: 10.1126/sciadv.adh5396 (PMC10516487; doi:10.1126/sciadv.adh5396)
Supplement: Supplementary file 1 — Sections S1 to S5 Figs. S1 to S13 [file sciadv.adh5396_sm.pdf]

Science Advances
AAAS

Supplementary Materials for

Noise resistant phase imaging with intensity correlation

Jerzy Szuniewicz *et al.*

Corresponding author: Radek Lapkiewicz, radek.lapkiewicz@fuw.edu.pl

Sci. Adv. **9**, eadh5396 (2023)
DOI: 10.1126/sciadv.adh5396

This PDF file includes:

Sections S1 to S5
Figs. S1 to S13

S1: Statistical model of the experiment

Two cameras are set on the two outputs of the interferometer, and each of them consists of the same number of pixels (n_{pix}). The sample area with phase ϕ_i is imaged onto the pixel number i on both cameras. Only two photons are received per the stability time of the interferometer phase. A single measurement consists of the detection of these two photons. The output of the single measurement is a pair $(i_{+/-}, j_{+/-})$. Numbers i, j stand for the numbers of pixels in which photons were detected, whereas indices $+$ or $-$ indicate in which of the two outputs the corresponding photon was measured. The probability of measuring a single photon in a pixel $i_{+/-}$ is:

$$p(i_{+/-}, \Theta) = \tilde{\mathcal{N}} I_i \frac{1}{2}(1 \pm v \cos(\phi_i + \Theta)), \tag{1}$$

where $\tilde{\mathcal{N}}$ is a normalization factor, v is interferometer visibility, Θ is an extra, spatially uniform, possibly fluctuating phase, and I_i is the intensity of the beam illuminating the phase mask in the area corresponding to pixel i. Phase Θ is stable within the time of detection of a single photon pair, its value for each pair is independently drawn from the continuous uniform probability distribution $p(\phi) = \frac{1}{2\pi}$ for $\phi \in [0, 2\pi]$. We do not have access to the randomly chosen value of Θ, so the observed probability of measuring pair $(i_{+/-}, j_{+/-})$ in every single frame is:

$$p(i_{+/-}, j_{+/-}) = \int_0^{2\pi} p(i_{+/-}, j_{+/-}, \Theta) d\Theta, \tag{2}$$

where $p(i_{+/-}, j_{+/-}, \Theta) = p(i_{+/-}, \Theta) p(j_{+/-}, \Theta)$ is a joint probability distribution of measuring pair $(i_{+/-}, j_{+/-})$ with the fixed value of Θ. From Eq. 2, we obtain the formulas:

$$p(i_+, j_+) = p(i_-, j_-) = 2\mathcal{N} I_i I_j \left(1 + \frac{v^2}{2} \cos(\phi_i - \phi_j)\right) \tag{3}$$

$$p(i_+, j_-) = p(i_-, j_+) = 2\mathcal{N} I_i I_j \left(1 - \frac{v^2}{2} \cos(\phi_i - \phi_j)\right), \tag{4}$$

where $\mathcal{N}$ is a new normalization factor. Notice, that Eq. (3) from the main text is retrieved from the two above equations after substituting $v = 1$ and neglecting the

normalization factor. The above equations are the starting point for further inference about the maximal precision of the measurement. Full information about every single measurement is included in the dependence of the probability $p(i_\pm, j_\pm)$ on the estimated parameters ϕ_i.

S2: Fundamental precision limits in interferometric phase imaging

S2.1 Rapid fluctuations regime

In order to calculate the maximal precision of estimation of the parameters ϕ_i, Fisher Information (FI) matrix F will be calculated. Then the inverse of the covariance matrix for all sets of unbiased estimators $\tilde{\phi}_i$ is lower bounded by the inverse of FI matrix [43]. There are 4 different types of events, which can occur during one experiment - two photons may be detected in one output (+ or $-$) or in different outputs (we distinguish between $+-$ and $-+$). We can distinguish between these 4 types, so the FI is the sum of FI matrices for all events types:

$$F = F^{++} + F^{--} + F^{+-} + F^{-+} \tag{5}$$

From equations 3 and 4 we can simply conclude, that $F^{++} = F^{--}$ and $F^{+-} = F^{-+}$. Let us now focus on the calculation of F^{++} matrix.

In order to simplify the formulas, the following notation will be used:

$$p(i_+, j_+) \equiv p(i,j), \quad \frac{\partial}{\partial \phi_k} \equiv \partial_k$$

The elements of the F^{++} can be written in the following form:

$$F_{kl}^{++} = \sum_{i,j=1}^{n_{\mathrm{pix}}} \frac{\partial_k p(i,j) \partial_l p(i,j)}{p(i,j)}, \tag{6}$$

Moreover,

$$\partial_k p(i,j) = \mathcal{N} I_i I_j v^2 (\delta_{jk} - \delta_{ik}) \sin(\phi_i - \phi_j), \tag{7}$$

$$\partial_k p(i,j) \partial_l p(i,j) = (\delta_{jk} - \delta_{ik})(\delta_{jl} - \delta_{il}) \mathcal{N}^2 I_i^2 I_j^2 v^4 \sin^2(\phi_i - \phi_j), \tag{8}$$

where δ_{ij} is a Kronecker delta. Consequently,

$$F_{kl}^{++} = \sum_{i,j=1}^{n_{\mathrm{pix}}} \frac{(\delta_{jk} - \delta_{ik})(\delta_{jl} - \delta_{il}) \mathcal{N} I_i I_j v^4 \sin^2(\phi_i - \phi_j)}{2 + v^2 \cos(\phi_i - \phi_j)} \tag{9}$$

If $k \neq l$, then for any m we have $\delta_{mk}\delta_{ml} = 0$, so $(\delta_{jk} - \delta_{ik})(\delta_{jl} - \delta_{il}) = -\delta_{jk}\delta_{il} - \delta_{ik}\delta_{jl}$. That means, that non-diagonal matrix elements are:

$$F_{kl}^{++} = -\frac{2\mathcal{N} I_k I_l v^4 \sin^2(\phi_k - \phi_l)}{2 + v^2 \cos(\phi_k - \phi_l)}, \quad k \neq l \tag{10}$$

With the help of the equality $(\delta_{jk} - \delta_{ik})^2 = \delta_{jk} + \delta_{ik} - 2\delta_{ik}\delta_{jk}$ we can obtain diagonal terms of F^{++}:

$$F^{++}_{kk} = 2\mathcal{N} I_k v^4 \sum_{i=1}^{n_{\mathrm{pix}}} \frac{I_i \sin^2(\phi_i - \phi_k)}{2 + v^2 \cos(\phi_i - \phi_k)} \tag{11}$$

For any function f:

$$\sum_{i=1}^{n_{\mathrm{pix}}} f(\phi_i, I_i) = n_{\mathrm{pix}} \langle f(\phi_i, I_i) \rangle_i, \tag{12}$$

where $\langle f(\phi_i, I_i) \rangle_i$ is the mean value of the function over all pixels. From now on, we assume that the number of pixels is big and that each phase in the sample occurs with the same frequency. What is more, the intensity of illuminating beam I_i is assumed to change slowly compared to the change of phase ϕ_i. In other words, many different phases occur in the region with approximately constant intensity. From these assumptions, we obtain the equality

$$\langle f(\phi_i, I_i) \rangle_i = \frac{1}{2\pi} \int_0^{2\pi} f(\phi, \langle I \rangle) d\phi, \tag{13}$$

which is true when f is linear with its second argument I, $\langle I \rangle$ stands for the mean intensity of the illuminating beam. Using the above assumptions, we can rewrite equation 11 as:

$$F^{++}_{kk} = 2\mathcal{N} I_k \langle I \rangle v^4 \frac{n_{\mathrm{pix}}}{2\pi} \int_0^{2\pi} \frac{\sin^2(\phi - \phi_k)}{2 + v^2 \cos(\phi - \phi_k)} d\phi \tag{14}$$

Consequently, diagonal terms of F^{++} are:

$$F^{++}_{kk} = 2\mathcal{N} \langle I \rangle I_k n_{\mathrm{pix}} (2 - \sqrt{4 - v^4}) \tag{15}$$

Let us now calculate the normalization factor $\mathcal{N}$. We will use the fact, that sum of probabilities of all events must be equal to one:

$$\sum_{i,j=1}^{n_{\mathrm{pix}}} p(i_+, j_+) + p(i_+, j_-) + p(i_-, j_+) + p(i_-, j_-) = 1 \tag{16}$$

Using equations 3 and 4 we obtain:

$$8\mathcal{N} \sum_{i,j=1}^{n_{\mathrm{pix}}} I_i I_j = 1 \tag{17}$$

We can rewrite the sum in the above equation as:

$$\sum_{i,j=1}^{n_{\mathrm{pix}}} I_i I_j = \left(\sum_{i=1}^{n_{\mathrm{pix}}} I_i \right)^2 = n^2_{\mathrm{pix}} \langle I \rangle^2 \tag{18}$$

and obtain:

$$\mathcal{N} = \frac{1}{8 n^2_{\mathrm{pix}} \langle I \rangle^2} \tag{19}$$

Finally, $^{++}$ matrix can be written in the form:

$$F_{kl}^{++} = \begin{cases} \frac{1}{4n_{\text{pix}}} \frac{I_k}{\langle I \rangle} (2 - \sqrt{4 - v^4}) & \text{for } k = l \\ \\ -\frac{1}{4n_{\text{pix}}^2} \frac{I_k I_l}{\langle I \rangle^2} \frac{2v^4 \sin^2(\phi_k - \phi_l)}{2 + v^2 \cos(\phi_k - \phi_l)} & \text{for } k \neq l \end{cases} \tag{20}$$

We have calculated the F^{++} matrix, which is obviously the same as F^{--} matrix, because formulas for probabilities in both cases are the same. Analogous calculation show, that also $F^{+-} = F^{-+} = F^{++}$. Using the FI additivity we obtain the terms of F matrix:

$$F = 4F^{++} \tag{21}$$

This is the FI matrix associated with the measurement of a single frame. If the total number of n_{phot} photons is detected in the experiment (which means $n_{\text{phot}}/2$ independent photon pairs), then, from the Cramér-Rao (C-R) bound,

$$\Delta^2 \phi_k \geq \frac{1}{n_{\text{phot}}/2} (F^{-1})_{kk} \tag{22}$$

In general, the estimator which satisfies the above inequality may not exist, however, it is possible to get arbitrarily close to the above bound if the number of measurements is big enough. That means, that the inequality becomes an equality if $n_{\text{phot}} \to \infty$. To simplify the calculations we also use the inequality:

$$(F^{-1})_{kk} \geq (F_{kk})^{-1}, \tag{23}$$

which is true for all hermitian F. The above inequality is not saturated in general, especially when non-diagonal terms of F are significant. However, in our case, the non-diagonal terms are asymptotically n_{pix} times smaller than diagonal terms. n_{pix} is also size of the F matrix. It may be proven, that for such scaling of non-diagonal terms with the size of the matrix, the above inequality becomes saturable for $n_{\text{pix}} \to \infty$. Using both of the above inequalities, we obtain the following bound:

$$\Delta \phi_k \geq \sqrt{\frac{n_{\text{pix}} \langle I \rangle}{n_{\text{phot}} I_k}} \frac{1}{\sqrt{1 - \sqrt{1 - v^4/4}}} \tag{24}$$

The value $n_k = \frac{n_{\text{phot}} I_k}{n_{\text{pix}} \langle I \rangle}$ may be interpreted as the expected number of photons detected in pixel number k. The above bound may be rewritten in the intuitive form:

$$\Delta \phi_k \geq \sqrt{\frac{1}{n_k}} \frac{1}{\sqrt{1 - \sqrt{1 - v^4/4}}} \tag{25}$$

From this form of inequality, it's clear, that the accuracy of measuring the value of the particular phase depends directly on the number of photons interacting with the measured area.

S2.2 Comparison with slow fluctuations regime

Let's compare our result with the phase estimation precision limit for an interferometer with a slowly fluctuating phase Θ. First of all, let's notice that we can't beat the accuracy achievable in the situation, in which extra phase Θ is known for all the detected photons—the information we get in a situation with unknown Θ is always smaller, even if the stability time of the interferometer is bigger. If Θ values are known, each single photon detection could be treated as an independent event (which was not the case in the previous section). Let's calculate the FI matrix for the single photon detection when Θ is fixed. A single measurement is fully described by the probability distribution from equation 1, and

$$\partial_k p(i_{+/-}) = \mp\frac{1}{2}\delta_{ki}\tilde{\mathcal{N}} I_i v \sin(\phi_i + \Theta). \tag{26}$$

In the case with fixed Θ, the one-photon FI matrix is

$$F^{(1)}_{kl} = \sum_{i=1}^{n_{\text{pix}}} \frac{\partial_k p(i_+)\partial_l p(i_+)}{p(i_+)} + \sum_{i=1}^{n_{\text{pix}}} \frac{\partial_k p(i_-)\partial_l p(i_-)}{p(i_-)}. \tag{27}$$

From equation 26 it is clear, that all non-diagonal terms of the $F^{(1)}$ matrix are equal to zero. This is because we obtain information about the ϕ_i phase only in case of detection of a photon in the pixel $i_{+/-}$. The diagonal terms are

$$F^{(1)}_{kk} = \tilde{\mathcal{N}} I_i \frac{v^2 \sin^2(\phi_i + \Theta)}{1 - v^2\cos^2(\phi_i + \Theta)}. \tag{28}$$

To make this case similar to the case described in the previous section let's assume, that Θ fluctuates and each value of Θ appears with the same frequency (the difference is that Θ fluctuates slowly and we know its value). Then the mean FI for the single measurement is:

$$\langle F^{(1)}_{kk}\rangle_\Theta = \frac{1}{2\pi}\int_0^{2\pi} F^{(1)}_{kk} d\Theta = \frac{I_i}{n_{\text{pix}}\langle I\rangle}\left(1 - \sqrt{1 - v^2}\right), \tag{29}$$

where formula $\tilde{\mathcal{N}} = \frac{1}{n_{\text{pix}}\langle I\rangle}$ obtained from the normalization condition was used. If we define $n_k = \frac{n_{\text{phot}} I_k}{n_{\text{pix}}\langle I\rangle}$ as in the previous section, we obtain the best possible accuracy of measuring each phase ϕ_k:

$$\Delta\phi_k \geq \frac{1}{n_{\text{phot}}\sqrt{F^{(1)}_{kk}}} = \sqrt{\frac{1}{n_k}}\frac{1}{\sqrt{1 - \sqrt{1 - v^2}}} \tag{30}$$

Equation 30 is very similar to the equation 25- the only difference is that term $\frac{v^4}{4}$ is substituted by the term v^2. That means, that the fact that one has only two photons per phase fluctuations stability time, leads to a decrease of the effective visibility of the interferometer from v to $\frac{v^2}{2}$, compared to a slowly fluctuating case, in which we can assume, that we know the value of Θ.

S3: Experimental setup details

Our experimental setup comprises a polarization-based Michelson interferometer equipped with a $4f$ imaging system. As a light source, we use a diode laser at a wavelength of $780nm$ coupled to a single-mode fiber. At the output of the fiber, for intensity and polarization control, the beam passes through a half-wave plate, a quarter-wave plate, and polarizing beam splitter (PBS), and another half-wave plate, and then enters the interferometer. Each of the two paths in the interferometer is encoded with orthogonal polarization. In order to imprint different kinds of phase profiles $\phi(x)$ on the object beam, we build two kinds of slightly modified setups—one with a cylindrical lens placed in front of the mirror in the horizontally polarized light beam path in the interferometer, while in the other setup, we replace the mirror in the same path with a spatial light modulator (SLM). The interferometric mirror in the reference arm is given a phase fluctuation by attaching it to a piezoelectric actuator driven by a ramp function signal.

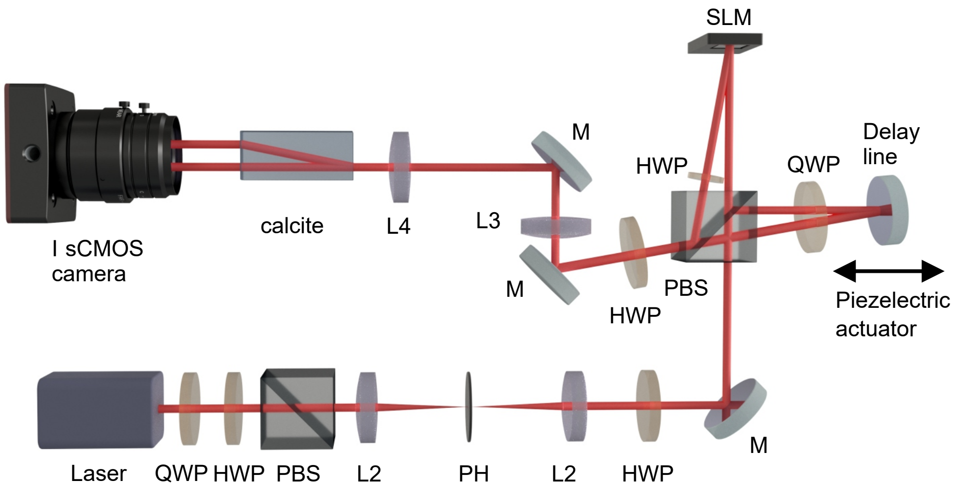

Figure 1: Experimental setup for noise-resistant phase imaging with SLM.

We perform experiments with three kinds of different phase masks applied to our object beam. Our first configuration is to imprint a one-dimensional quadratic local phase profile to the beam by placing a cylindrical lens of focal length, $f = 1000$ mm in proximity to the mirror Additionally, in our second configuration with SLM (from the HOLOEYE PLUTO) as a phase mask, we can display any arbitrary phase profile. As an example, we imprint one-dimensional exponential and sinusoidal phases onto our object beam. Since the SLM is efficient for only one polarization of light (in our case horizontal), we place the SLM in the horizontal path of the interferometer and the reflected beam with a given phase mask (the object beam) passes through a $\lambda/2$ plate. We introduce a time-dependent phase fluctuation $\Theta(t)$ in the reference arm - vertically polarized beam path in the interferometer) to make it effectively incoherent with the object beam. This is realized with a piezoelectric actuator driven by a ramp of 1.234 Hz. This shouldn't

be confused with the maximal noise frequency for which our method works. Both the object and reference beams are combined on the polarizing beam splitter (PBS). At the output of the interferometer, we have a 4f imaging system consisting of lenses L3 and L4 of focal length 200mm each to image the SLM plane (phase plane) the intensified sCMOS (I-sCMOS - with the image intensifier from Hamamatsu V7090D-71-G272 and sCMOS from Andor Zyla) camera. To observe the interference, the orthogonally polarized object and the reference beam are required to be indistinguishable, and to do so, we rotate the polarization of both beams by 45 degrees with a half-wave plate and we perform the projective measurement in the original bases with a calcite crystal. Here, the calcite along with the waveplate act as a 50/50 Beamsplitter. This mixes the light from both outputs and allows us to observe interference in both outputs of the splitter. The I-sCMOS camera records single photons at both outputs of the interferometer on two regions. In order to get a high interference visibility within a single frame, we keep the camera exposure time low such that fluctuations are negligible within one camera frame. The use of short exposure time of the I-sCMOS, in the single nanosecond timescale, gives it stability and resistance against fluctuations up to tens of MHz. The interference visibility is slightly reduced due to imperfect imaging because of the path length difference in the calcite. We collect the data with a 200 Hz of frame rate.

S4: Data analysis

In this section, we describe how to obtain the final phase profiles from the raw experimental data. We first describe a standard approach for a non-fluctuating phase Θ, which we treat as verification of the ground truth. Then, we will follow with the analysis of the phase-fluctuating case and quantitative analysis of the reconstruction precision.

S4.1 One dimensional non-fluctuating phase

For ground truth measurements, we stabilize the interferometer with a box to reduce the airflow in the setup and use a laser beam to record bright interference fringes in a single shot. We record these fringes using a standard CMOS camera (Matrix Vision mvBlueFOX-IGC). We are then blocking interferometer paths, one at a time, to record beam profiles. We use these profiles to normalize the interference fringes and get an interferogram not modulated by the input beam profile.

We add padding—increase the size of the image from n_{pix} by n_{pix} pixels to $3n_{\mathrm{pix}}$ by $3n_{\mathrm{pix}}$ pixels by adding an n_{pix} pixel ramp function on each side of the array from the image's edge value to zero (see NumPy library documentation for full description: numpy.pad, mode = 'linear__ramp', end_values = 0). Adding padding increases the available Fourier space and allows us to get a smoother phase retrieval. Then the normalized and padded interferogram is converted to the Fourier space using a fast Fourier transform (FFT). Then we extract half of the data, removing low frequencies in the middle. This is a common practice for phase retrieval [44] and allows us to directly retrieve the phase—by inversing the FFT we get the phase as an argument of complex numbers

from the resulting map.

The reconstructed phase is still wrapped—an argument lies in the range from 0 to 2π so the phase needs to be unwrapped; we perform this using the SciPy library in Python. At the end of the process, we get a two-dimensional phase profile, and since the analyzed phase is one-dimensional, we average the retrieved phase within the area of sufficient signal strength over one of the dimensions. We end up with a one-dimensional phase profile that was created on the basis of standard, well-established interferometric method and that can be treated as the basis for further experiments.

S4.2 One Dimensional Fluctuating Phase

For the creation of the joint probability map, we record the positions of all photons. We are considering only one-dimensional cases, so it is enough to know the position along the direction in which the phase changes. We extract the correct positions and create a square array with dimensions of n_{pix} by n_{pix}, where n_{pix} is the number of pixels rows for the single-photon camera. This square array is our correlation map—its element with coordinates (i, j) contains the number of frames in which a pair of photons, one at position i, another at position j, was detected. In principle, two photons detected per frame are enough to create such a correlation map, given a sufficient number of measured frames.

We create correlation maps for all frames and sum them. We finally get an average joint probability map that contains phase information $\phi(x)$:

$$\left\langle \tilde{I}(x;t)\tilde{I}(x\prime;t) \right\rangle \propto 1 \pm \frac{v^2}{2} \cos\left[\phi(x) - \phi(x\prime)\right], \tag{31}$$

where $v^2/2$ is the visibility of 2nd order intereferogram fringes. We use notation $v^2/2$ because in an ideal scenario, visibility of 1st order fringes v (observed for non-fluctuating case) would lead to visibility $v^2/2$ observed for fluctuating case for a 2nd order interference. Notice, that the above equation is equivalent to Eq. (3) from the main text—the difference is that now we consider 1D phase profile and imperfect interference visibility. Please note that at this point we can use exactly the same phase retrieval method as for the non-fluctuating case, as the phase information is imprinted in a form resembling the first-order interference: $\cos\left[\phi(x, y) - \phi(x\prime, y\prime)\right]$. We use the same data analysis as in Section 4.1 of this document. The only difference is that we normalize the joint intensity map using a sum of all interferograms, instead of the sum of separate measurements, as the phase information in the first-order interferogram is lost (please refer to the normalization steps in Fig. 2).

S4.3 Quantitive error analysis

We want to assess the accuracy of our phase imaging method in the most extreme scenario, in which only two photons are collected in each frame. Phase fluctuations are fast compared to the time between subsequent frames, but the fluctuating phase Θ is stable within a single frame—the same assumptions were made in S1 and S2.

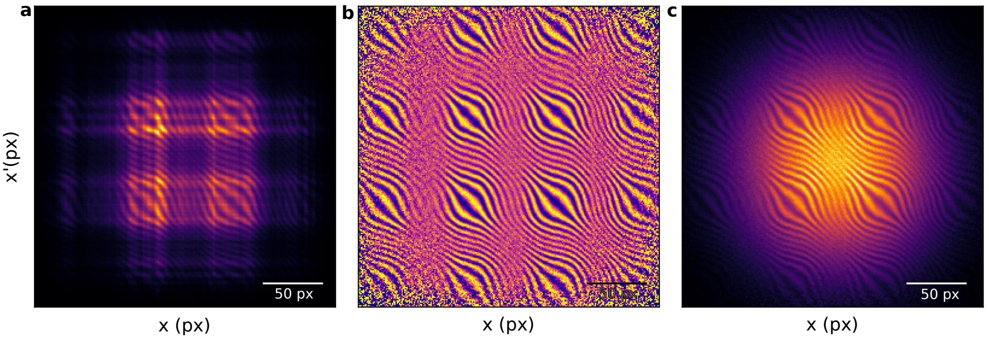

Figure 2: Additional steps for a highly distorted beam data processing: **a)** A raw coincidence map, created from all frames of the measurement. **b)** A normalized coincidence map in respect to the intensity of the beam. Even low photon count regions carry phase information. **c)** normalized coincidence map from (b) multiplied by the Gaussian profile of the perfect beam. We add this step for the off-axis Fourier analysis to remove high-noise regions and smoothing the interferogram. Please note, that despite raw image being highly distorted from imperfections of the SLM, our phase retrieval works effectively and the normalized coincidence map has a high visibility.

Firstly, we need to construct the figure of merit assessing the phase retrieval accuracy. Let us assume, that our goal is to estimate the phase profile of the part of the object corresponding to n pixels, whose numbers are $m, m+1, ..., m+n-1$. The real phase in pixel k is ϕ_k, its estimation is $\tilde{\phi}_k$. Then, the MSE associated with the phase estimation in this region is

$$\mathrm{MSE} = \left\langle \Delta^2 \phi_k \right\rangle = \frac{1}{n} \sum_{k=m}^{m+n-1} (\Delta\phi_k)^2 = \frac{1}{n} \sum_{k=m}^{m+n-1} (\phi_k - \tilde{\phi}_k)^2, \tag{32}$$

where the difference $\phi_k - \tilde{\phi}_k$ is always taken modulo 2π, such that $\phi_k - \tilde{\phi}_k \in [-\pi, \pi]$.

The theoretical minimum of the MSE can be obtained using C-R bound as described in S2—it is enough to insert Eq. 25 into Eq. 32. This theoretical minimum is depicted in Figure 2 (in the main text) using a solid, black line.

To check the optimality of our phase reconstruction method, we compare this minimum with the MSE obtained in the simulation. For a given beam profile I_i, phase profile ϕ_i and visibility v, we randomly sample $n_{\mathrm{phot}}/2$ pairs of x photons positions from the probability distribution described by Eq. 4. Therefore, in our simulation, there are only two sources of noise—non-unit interference visibility and shot noise, resulting from the finite number of photon pairs. The number of photon pairs is two times smaller than the total number of photons because one pair is created from each two-photon pair—this makes all pairs statistically independent. After creating an artificial hologram, we perform the whole phase reconstruction procedure described in section S4.2 to get phase estimators $\tilde{\phi}$. Then, we check a region of interest in the middle of the beam—we don't

want to take into account regions where the beam intensity is very low. Finally, we compute the MSE associated with the phase estimation in this region using Eq. 32. We repeat this procedure for different total number of photons n_{phot}, which leads to different mean number of photons per pixel. We can observe in Figure 3, that the performance of our phase reconstruction method is optimal for large enough number of photons.

We also perform a similar, quantitative analysis using experimental data. To make the comparison between theory, experiment and simulation possible, we measure the beam intensity, phase profile and the visibility of 2nd order interference fringes in the correlation map ($v^2/2$). Then, the experimental parameters are used in the simulation and to calculate the C-R bound. We obtain phase estimators $\tilde{\phi}_i$ using experimentally measured correlation map. To obtain such a map, we randomly choose just one photon pair from each frame—the goal is to simulate the experimental conditions, in which only two photons per frame are available. The ground truth phase profile ϕ_i is quadratic because we use a thin lens as a phase mask. The parameters of this ground truth phase profile are measured using conventional Fourier off-axis holography technique stable phases in both arms. The experimental phase estimators $\tilde{\phi}_i$ are calculated without any assumptions about the shape of the phase profile—we don't assume, that the measured phase profile is quadratic. The MSE for the region of interest is again computed using Eq. 32. We observe, that the MSE from experiment and simulation are very similar—they only start to differ slightly for the number of photons per pixel $\sim 4 \times 10^4$. This may suggest, that noise sources other than shot noise start to play a significant role at this point.

Let us now briefly discuss the fact that both simulation and experiment saturate the C-R bound only for the sufficiently high numbers of photons per pixel. The C-R bound is always a valid lower bound for the MSE that can be achieved in a given experiment for any unbiased estimator. However, the C-R bound is not always guaranteed to be saturated (even for the optimal choice of an estimator). There are, however, two cases in which it is always possible to find a C-R bound-saturating estimator:

1. When the estimated parameter is guaranteed to be very close to some known value, and we only want to estimate a small deviation from this value.

2. When statistically independent and identical measurements are performed N times, and N is large enough – strictly speaking, C-R bound is always saturated asymptotically for $N \to \infty$.

In our case, we do not have any initial knowledge about a phase pattern, so we are not working in a local estimation regime (1). Therefore, C-R bound is guaranteed to be saturated only in case (2), so for large N. The fact that the MSE is much larger than the C-R bound for a small number of repetitions is typical, especially for multi-parameter estimation, as is the case in our experiment. The mathematical tools that allow to calculate bounds that are saturable for small N are much more complicated and usually require some additional assumptions about a priori knowledge about the estimated quantity. That's why C-R bound is so common in literature, even though it doesn't properly capture the behavior of the MSE for small N.

In our particular case, the off-axis holography method fails when the noise is too large, as then it is no longer possible to separate large and small spatial frequencies. Consequently, the C-R bound starts to be saturated when the amount of collected signal is large enough.

S5: Two Dimensional Phase Measurement and Simulation

In this section, we describe how to retrieve information about 2D phase patterns from an intensity correlation map. Both simulation and experiment confirm, that our technique works properly also for 2D phase imaging.

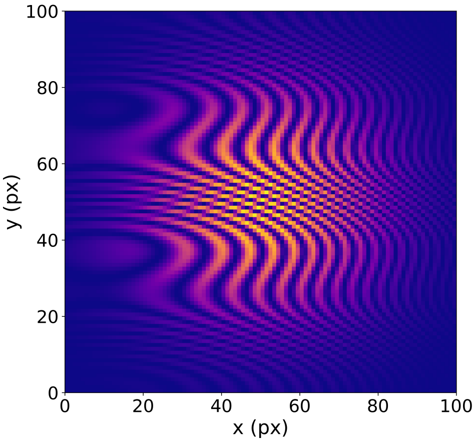

Figure 3: Simulated, perfect noise-free, interferogram with a spatial phase from eq. (35), which is the combination of the quadratic phase in x and the sinusoidal phase in y. An extra linear phase was added in order to enable the off-axis holography phase retrieval method

S5.1 From 2D camera frames to 4D correlations

The fundamental difference between 2D and 1D phase imaging is the dimensionality of the created correlation map: a 2D phase measurement results in a 4D correlation map, while a 1D phase requires a 2D correlation map. During the experiment, we measure intensity distributions at the two outputs of the interferometer ($\tilde{I}(x, y; t)$ and $\tilde{I}(x', y'; t)$ respectively), and then create an intensity product map $C_t(x, y, x\prime, y\prime; t)$ for each time frame (labeled by t):

$$C_t(x, y, x\prime, y\prime; t) = \tilde{I}(x, y; t) \cdot \tilde{I}(x\prime, y\prime; t), \tag{33}$$

To decrease the shot noise, we average all intensity product maps $C_t(x, y, x\prime, y\prime; t)$

created for subsequent time frames to get an averaged correlation map

$$C(x,y,x\prime,y\prime) = \left\langle \tilde{I}(x,y;t)\tilde{I}(x\prime,y\prime;t) \right\rangle \propto 1 \pm \frac{1}{2}\cos\left[\phi(x,y) - \phi(x\prime,y\prime)\right], \qquad (34)$$

where $\phi(x,y)$ is the phase at the position x, y of a measured sample (see also Eq.(3) in the main text). It is also possible to only use a single interferometer output $\tilde{I}(x,y;t)$ and correlate it with itself – this results in changing the sign in eq. (34). Since we average 2nd-order correlation maps, not intensities, we can arbitrarily reduce the shot noise without affecting the visibility of fringes. Eq. (34) is valid for the interferometric phase $\Theta(t)$ fluctuations which completely wash out first-order interference fringes (which happens when each value of $\Theta(t)$ between 0 and 2π appears with the same probability). As already discussed in the main text, this condition can be realized by adding an extra, slowly varying, phase noise to the setup—one can easily add such noise to an interferometric arm in the experiment (for instance with a piezoelectric actuator).

S5.2 Perfect correlation map

In this section, we will be analyzing a simple 2D phase mask:

$$\phi(x,y) = \frac{x^2}{20} + 10\sin\left(\frac{y}{3}\right), \qquad (35)$$

that is a sum of the quadratic phase in x and the sinusoidal phase in y. An exemplary 1st order interferogram for the given spatial phase and a Gaussian beam intensity is presented in Fig.3. We add an extra linear phase to both x and y axis of the phase $\phi(x,y)$ to use standard off-axis Fourier holographic phase retrieval methods [44].

2D cross-sections of the 4D correlation maps:

Let us now focus on specific 2D cross-sections of the 4D correlation map C.

In order to visualize a 4D correlation map $C(x,y,x',y')$, we create its 2D cross-section. The simplest way of doing this is to fix two out of four parameters x,y,x',y'. For example, when we fix $x = x_0$ and $y = y_0$, we get a 2D cross-section $C_{x_0,y_0}(x',y') = C(x = x_0, y = y_0, x', y')$. We are going to use a similar short-hand notation for cross-sections with other fixed parameters, e.g. $C_{x_0,x'_0}(y,y') = C(x = x_0, y, x' = x'_0, y')$, etc. Examples of such cross-sections are presented in Fig.4 and Fig. 5.

1D cross-sections of the 4D correlation maps:

By fixing both primed and not primed dimensions for the same axis in equation (34) we obtain 2D correlations the same as for extensively discussed 1D phase measurements (see Supplement S4):

$$C_{y_0,y'_0}(x,x') \propto 1 \pm \frac{1}{2}\cos\left[\phi_{y_0}(x) - \phi_{y'_0}(x')\right], \qquad (36)$$

$$C_{x_0,x'_0}(y,y') \propto 1 \pm \frac{1}{2}\cos\left[\phi_{x_0}(y) - \phi_{x'_0}(y')\right], \qquad (37)$$

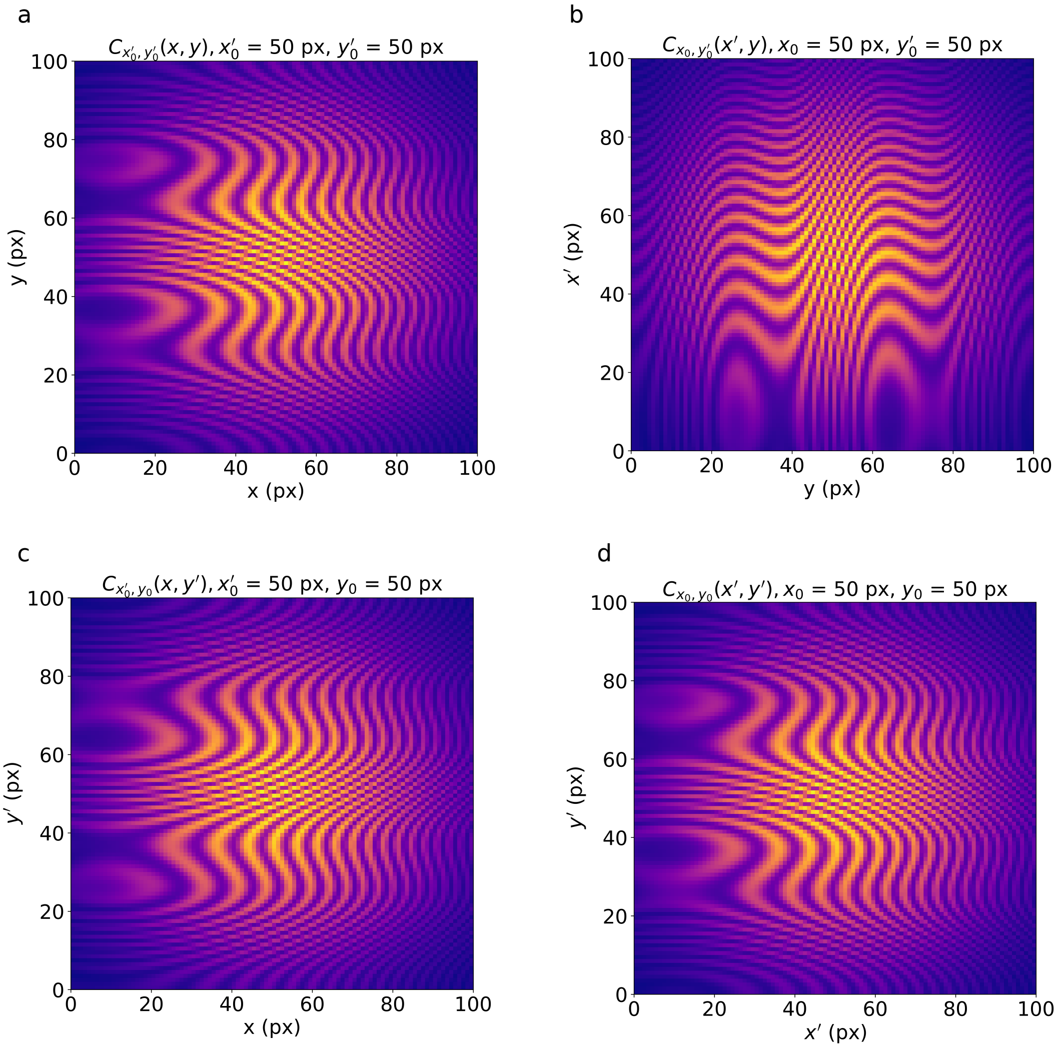

Figure 4: Two-dimensional correlation maps for simulated perfect data. **(a-d)** show correlation maps with four exemplary cross-sections where the spatial phase is a combination of the quadratic phase in x and the sinusoidal phase in y: $C_{x'_0,y'_0}(x,y)$, $C_{x_0,y'_0}(x',y)$, $C_{x'_0,y_0}(x,y')$, and $C_{x_0,y_0}(x',y')$.

where $\phi_{y_0}(x) = \phi(x, y = y_0)$ and $\phi_{x_0}(y) = \phi(x = x_0, y)$ are 1D slices of the initial phase $\phi(x, y)$ for $y = y_0$ and $x = x_0$ respectively. Please note, that in order to get the same correlation maps as for 1D phases we need to set $y_0 = y'_0$ for the former and $x_0 = x'_0$ for the latter case—only then, the resulting correlation map is created using the same line of the 2D phase $\phi(x, y)$. The corresponding 1D phase slices for axis x and y are presented in Fig.5, note that for x axis phase we can recognize the quadratic phase correlation map,

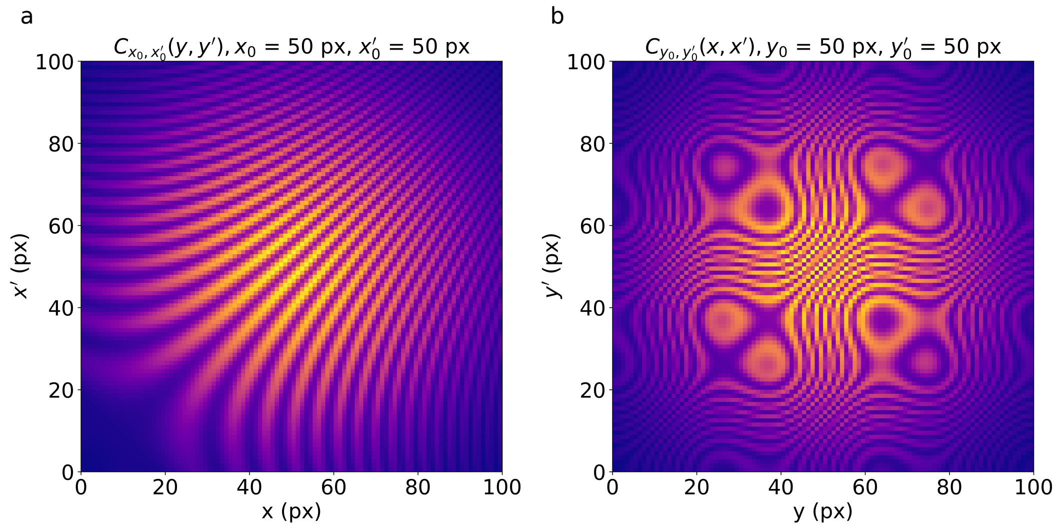

Figure 5: Correlation map cross-sections for 1D phase sub-spaces for simulated perfect data (no-noise). **(a)** Cross-sections for x axis: $C_{y_0,y_0'}(x,x')$; **(b)** cross-sections for y axis: $C_{x_0,x_0'}(y,y')$.

as in our case (Eq. 35):

$$\phi_{y_0}(x) = \frac{x^2}{20} + c(y_0), \tag{38}$$

where $c(y_0)$ is a constant not depending on x. The same applies to the y axis phase, which is sinusoidal:

$$\phi_{x_0}(y) = 10\sin\left(\frac{y}{3}\right) + c(x_0). \tag{39}$$

In the remaining part of this supplement, we will no longer focus on particular 2D cross-sections of the 4D correlation map—we will introduce an algorithm to retrieve the 2D phase using all the information carried by the 4D correlation maps.

S5.3 4D Correlation Map Analysis

The phase term in Eq. (34),

$$\Phi(x,y,x\prime,y\prime) = \phi(x,y) - \phi(x\prime,y\prime), \tag{40}$$

contains the full information about the spatial phase $\phi(x,y)$ and can be retrieved from $C(x,y,x',y')$ using standard off-axis Fourier holographic phase retrieval methods [44].

In practice, we first create a normalized correlation map,

$$\bar{C}(x,y,x\prime,y\prime) = \frac{C(x,y,x\prime,y\prime)}{\bar{I}(x,y)\bar{I}(x\prime,y\prime)}, \tag{41}$$

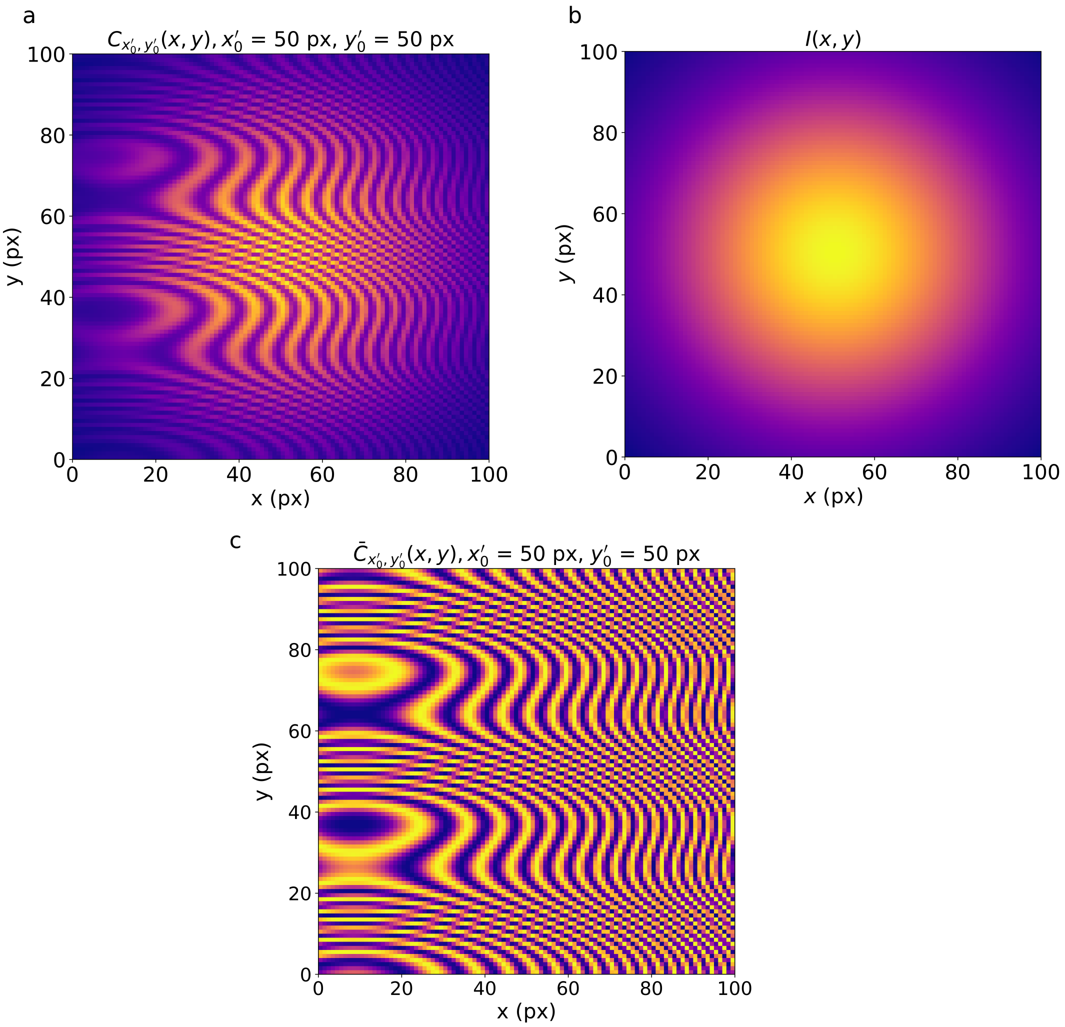

Figure 6: Correlation map through x and y subspace before and after normalization and the average intensity profile for simulated perfect data. **(a)** Presents the middle 2D slice of a simulated correlation map C, $(x'_0, y'_0) = (50, 50)$; **(b)** shows average intensity $I(x, y)$ measured at the output, contains no phase information; **(c)** is another 2D slice of C, but this time x, y are fixed.

where

$$\bar{I}(x,y) = \frac{1}{N_{x\prime}N_{y\prime}} \sum_{x\prime}\sum_{y\prime} C(x,y,x\prime,y\prime) \tag{42}$$

is the beam intensity profile, $N_{x\prime}$ and $N_{y\prime}$ are the numbers of camera pixels along the dimensions x and y respectively. $\bar{I}(x\prime, y\prime)$ is defined analogously.

We can now retrieve phase Φ from the normalized 4D correlation map $\bar{C}(x, y, x\prime, y\prime)$

using NumPy library fftn function that computes the n-dimensional fast Fourier transform (FFT). We replace half of the calculated Fourier matrix with zeros and remove the low-frequency parts (center and axes) which contain offset information about the DC (non-oscillating) part of the correlation map.

After filtering and masking in the Fourier space we transform the matrix back to the position space using the inverse FFT implemented with NumPy.ifftn function. As a result, we obtain the following 4D matrix:

$$\Psi(x, y, x\prime, y\prime) = O(x, y, x\prime, y\prime)\mathrm{e}^{i\Phi(x,y,x\prime,y\prime)}, \tag{43}$$

where we neglected the noise contribution. Note that, here we are not interested in $O(x, y, x\prime, y\prime)$, as we only want to retrieve the phase $\Phi(x, y, x\prime, y\prime)$. In order to retrieve the 4D phase matrix $\Phi(x, y, x\prime, y\prime)$ from Eq. 43 we take the argument of each element of $\Psi(x, y, x', y')$:

$$\tilde{\Phi}(x, y, x\prime, y\prime) = \arg\left(\Psi(x, y, x\prime, y\prime)\right). \tag{44}$$

As a result, we obtain $\tilde{\Phi}(x, y, x\prime, y\prime)$, which for a noiseless correlation map is equal to $\Phi(x, y, x', y')$ modulo 2π—$\tilde{\Phi}(x, y, x\prime, y\prime) \in [0, 2\pi]$, which means that the phase is wrapped. In order to unwrap it, we use Python library unwrap (https://pypi.org/project/unwrap/), that is based on [45].

The algorithm for phase unwrapping described in [45] can be only applied to 2D phase arrays, therefore we analyze all cross-sections of $\Phi(x, y, x', y')$ separately, iterating through all of the x' and y' values. As a result, we obtain $N = N_{x\prime} \cdot N_{y\prime}$ 2D phase matrices $\phi(x, y) + c(x\prime, y\prime)$, which differ by phase offset $c(x\prime, y\prime) = -\phi(x\prime, y\prime)$, constant within one slice.

After the Fourier analysis, some of the measured 2D phase arrays $\phi(x, y)$ for particular slices $x\prime, y\prime$ may contain phases that are hard to unwrap – such a matrix will contain many π phase jumps and can be flagged by creating the histogram of values within the wrapped phase matrix $\tilde{\phi}$. We then compute the ratio of 20 % of the highest counts in the histogram and based on that remove frames with highly non-uniform values distribution—we remove up to a few percent of all measured phase arrays. After cleaning the unwrapped maps we align them at a selected point—this removes the offset $c(x\prime, y\prime)$ from $\phi(x, y) + c(x\prime, y\prime)$. The choice of the point can be arbitrary, we choose the middle of the phase matrix, but for the most reliable comparison between actual and retrieved phase, we should choose the point of the highest intensity, as phase retrieval error at that point should be the smallest. Such aligned phase arrays can now be averaged to get the accurate estimate of the phase $\phi(x, y)$.

With such analysis described above, we utilize all the information present in the correlation map C, no information is lost. Combining this fact with the derived proof of optimality for correlation analysis (two photons per frame case) presented in Supplement 2, we can state that this method extracts all the phase information that is available.

We present the analysis of the perfect (noiseless) correlation map corresponding to the phase profile described by (35) in Fig.6. The phase is retrieved with a very high accuracy, minor systematic errors (~ 0.001rad) are caused by imperfect filtering of small frequencies in off-axis holography. In the following section, we are going to present the

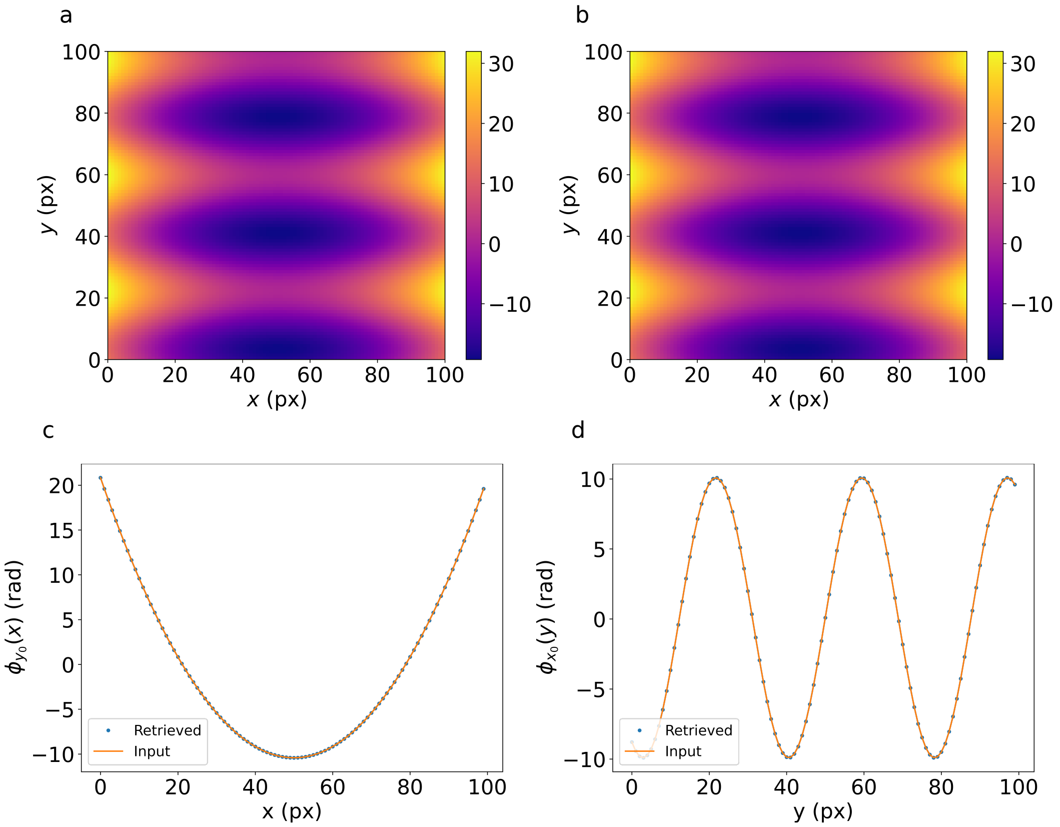

Figure 7: Phase reconstruction for simulated perfect data (no-noise); $\phi(x, y) = \frac{x^2}{20} + 10\sin\left(\frac{y}{3}\right)$. **(a–b)** show the introduced 2D phase $\phi(x, y)$ and the retrieved phase; **(c–d)** represent 1D averages of measured 2D phase along the x and y axes, and comparison with introduced phase.

simulation of the performance of our technique in more realistic conditions, when shot noise is also present.

S 5.4 Noisy simulated correlations

We analyze here simulated data for spatially-resolved single-photon detections. Correlation maps can be created from such measurements and averaged to a 4D correlation matrix C. Our analysis is made for bio-inspired, toroidal phase profile, shown in Fig. 10a and described by the following equation

$$\phi(x, y) = \max\left\{\frac{r^2 - (\sqrt{(x/1.2)^2 + y^2} - R)^2}{10}, 0\right\}. \tag{45}$$

It is worth noticing, that our method works even though the shape contains a sharp edge at $\phi = 0$, which may be the source of technical difficulties associated with off-axis Fourier

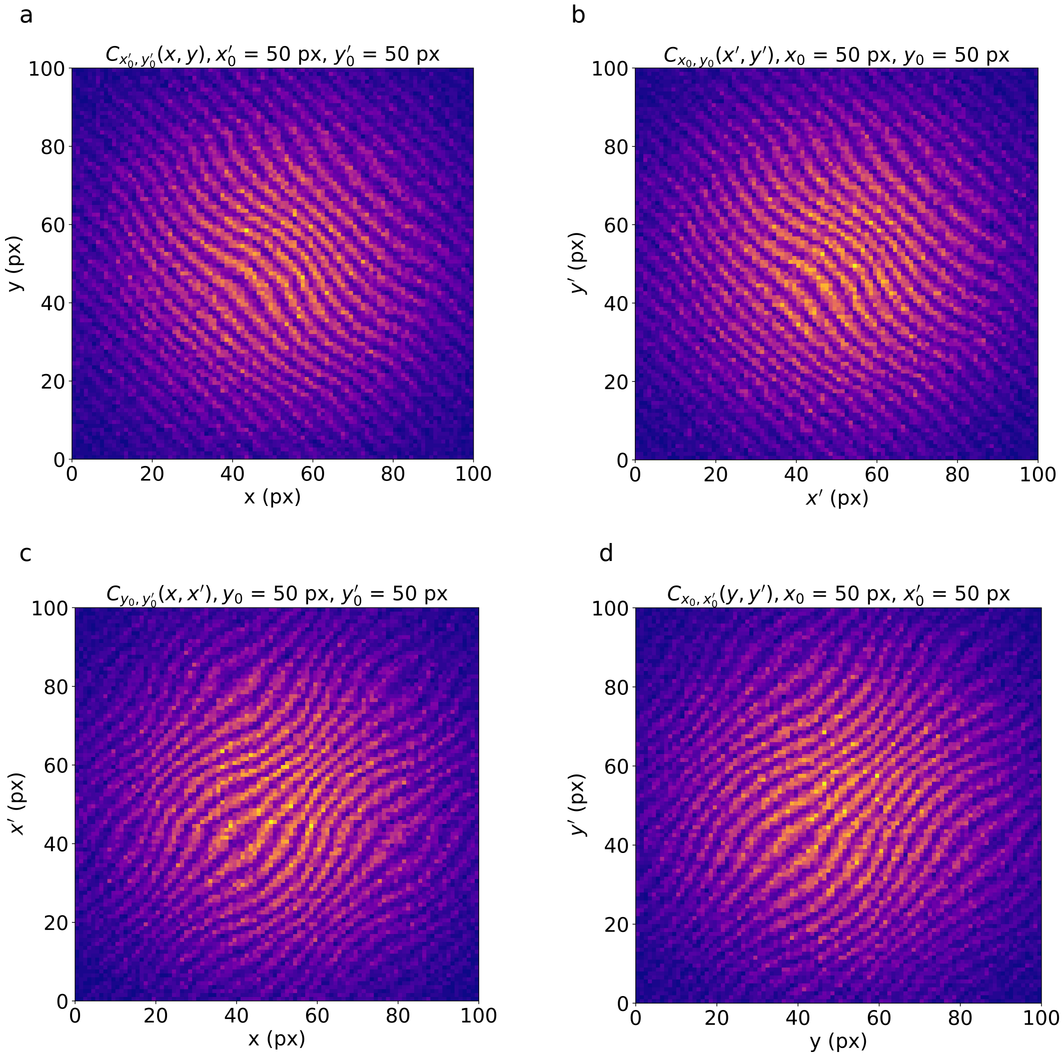

Figure 8: Correlation maps C for noisy simulated data. **(a)** shows a slice through not primed subspace $C_{x'_0,y'_0}(x,y)$; **(b)** a slice through primed subspace $C_{x0,y0}(x',y')$; **(c)** a slice through x subspace $C_{y0,y'_0}(x,x')$, corresponding to the phase $\phi_{y0,y'_0}(x,x')$ and **(d)** a slice through y subspace $C_{x0,x'_0}(y,y')$, corresponding to the phase $\phi_{x0,x'_0}(y,y')$.

analysis. The phase profile is discretized and represented as a 100×100 array (same size as in the experiment)

To simulate the shot noise, we first create a normalized, smooth map $\bar{C}(x,y,x',y')$. Then, each element of the noisy map is randomly drawn from the Poisson distribution with mean $n_c\bar{C}(x,y,x',y')$, where n_c is the expected total number of observed coincidences (photon pairs), for this analysis $n_c = 5 \cdot 10^8$ coincidences.

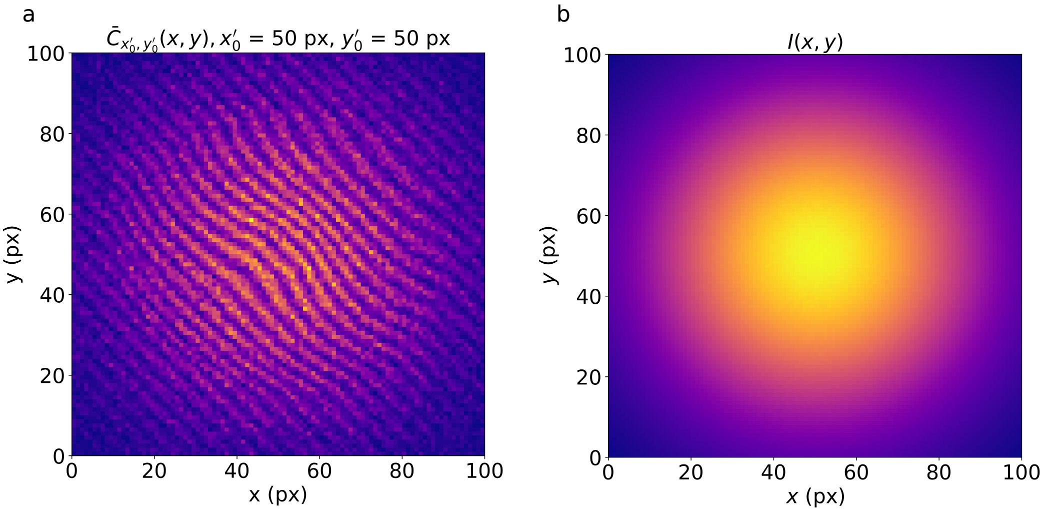

Figure 9: Normalized correlation map through x and y subspace and the average intensity profile for noisy simulated data. **(a)** Shows a single slice through the normalized correlation map $C_{x'_0,y'_0}(x,y)$, displaying x and y subspace. **(b)** Represents an average of (C) over x and y sub-spaces – the average of all frames in the experiment. The average contains no fringes.

We see that phase was retrieved well (Fig. 10b), especially taking into account the low signal regime, with an average value of absolute error across the middle 400 pixels square area of: 0.29 Radians, and a corresponding standard deviation of 0.8 Rad. For this calculation, we used 15% of all slices, which corresponded to the highest photon count areas and which were not causing unwrapping errors. We also performed simulations for a larger number of coincidences (which were not presented here) and concluded that the phase error drops with the increased signal levels as expected. It is also worth noting, that the uncertainty (standard deviation) of the phase is inversely proportional to the number of coincidences for a given pixel. For this reason, we choose to analyze only correlations with pixels containing enough signal – thanks to the multi-dimensionality of the correlation map we still obtain the full area of the phase measured. Fig. 10c shows the standard deviation of the phase retrieval obtained through analysis of all aligned phase maps.

We also show a direct comparison of 1D slices through the measured and introduced phase at different positions of y in Fig.11(a–d), showing good agreement in the higher beam intensity area. In summary, we demonstrated that the simulated data can be analyzed with our method and result in good retrieval of the measured phase.

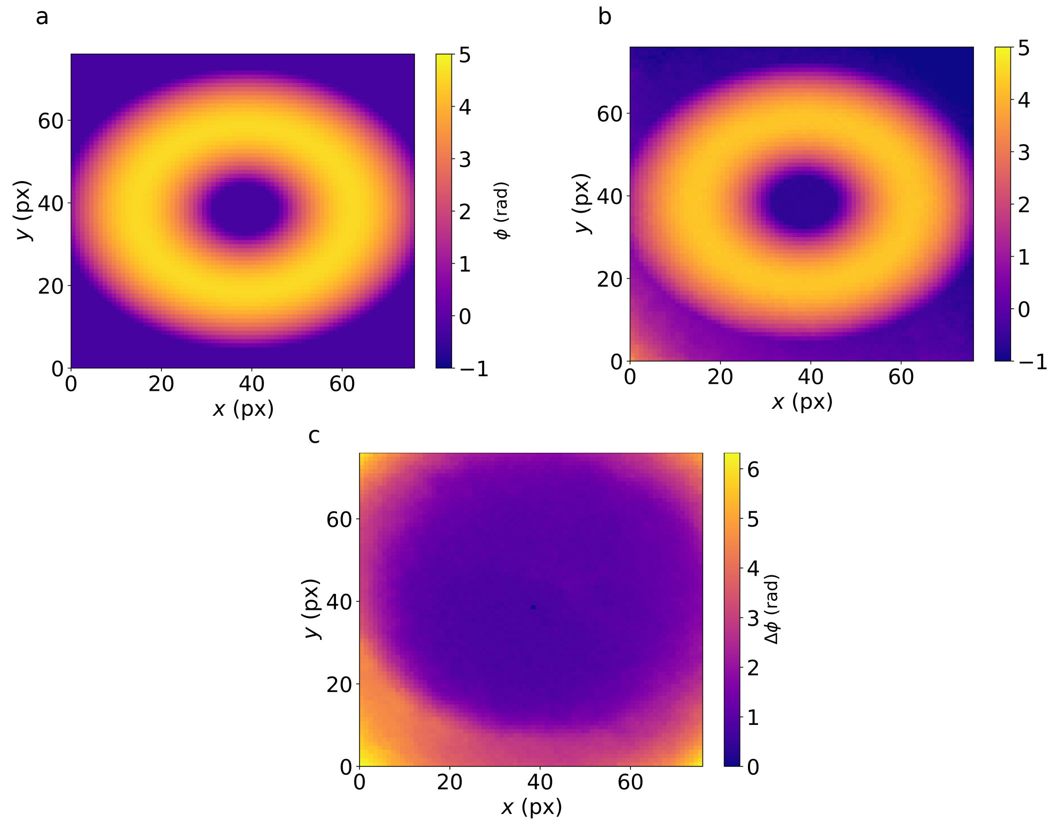

Figure 10: Phase reconstruction for the noisy simulated data. **(a)** Shows the introduced phase $\phi(x, y)$, **(b)** the retrieved phase, and **(c)** the standard deviation of the phase retrieval, which was obtained through analysis of all aligned phase maps.

S 5.5 2D Phase Experimental results

In this section, we present a full analysis of the 2D phase retrieval experiment from the main text. We decided to gather the imaging data with a standard CMOS camera, which is not single-photon counting. Using CMOS camera and keeping the illumination level (brightness of the interferogram) equal to or below the noise level of the camera prevents us from recording interference fringes directly – no beam or fringes are visible on any of the recorded frames (an exemplary, random, frame from the experiment is presented in Fig.12(a)). By proving the applicability of our method for phase retrieval of very noisy frames from a standard CMOS camera we drastically increase the applicability of our method - a single photon sensitive camera is optional and we can retrieve the correlation signal that was buried in the readout noise.

We used the same setup described in Supplement 3 and introduced external interferometric phase noise using a piezo actuator displacing one of the interferometer's mirrors. We introduced spatially dependant quadratic phase $\phi(x, y)$ using a standard optical lens

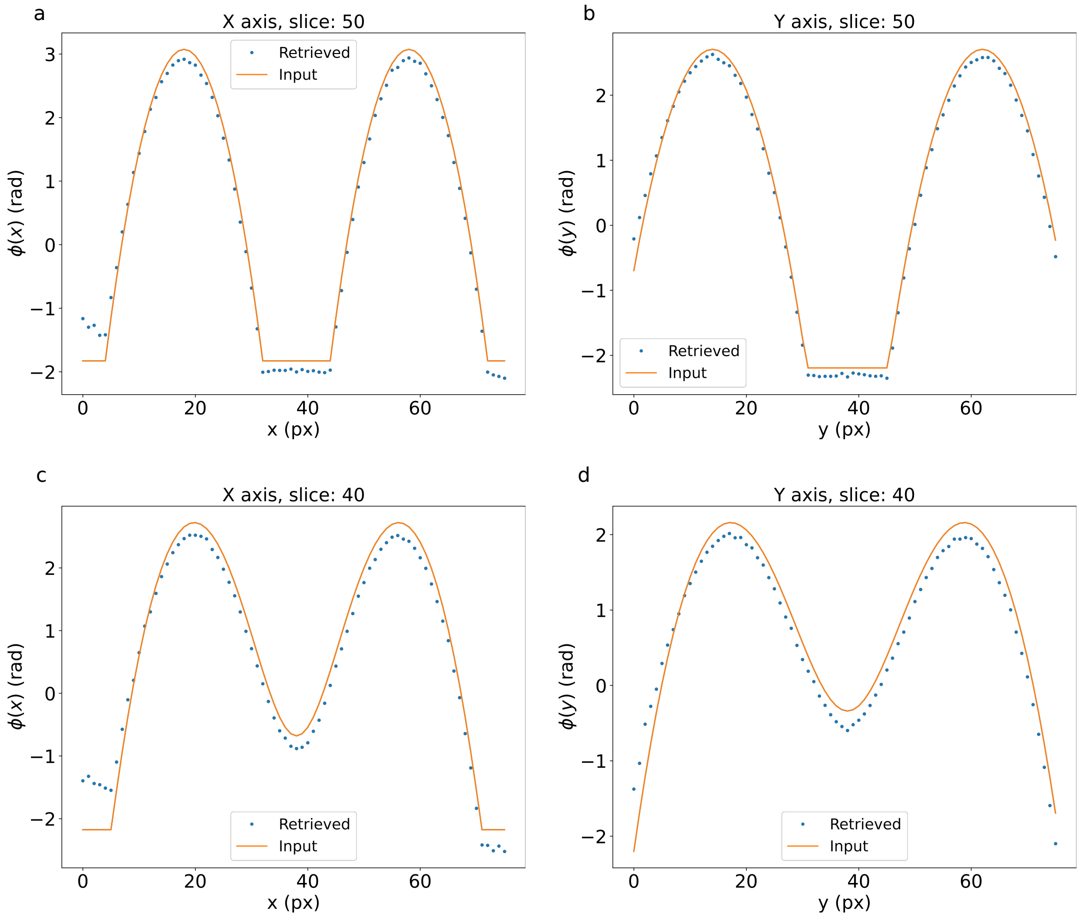

Figure 11: Comparison of measured and introduced 1D phase slices for noisy simulated data. **a–d** Represents comparison of introduced and measured 1D phase slices at different positions of x and y.

(Part number - LA1708-B-ML - Ø1" N-BK7 (Thorlabs) and focal length, f = 200 mm, ARC: 650 - 1050 nm). Fig.12(b) presents an average overall recorded frames (135 854 frames) – no fringes from interference are visible, however, there are fringes caused by different readout noise of analog-digital converters (ADC) of the CMOS camera. Since our signal has a lower amplitude than noise, we filter the image by dividing each row by its corresponding average value.

We calculate the correlation map and analyze the phase following the steps described in S5.2 and S5.3. Please note, that the unnormalized correlation map will suffer from the ADC noise of the camera, while the normalized will have most artifacts removed. The only modification of the algorithm is removing a high autocorrelation signal caused by the low signal level and high noise of the CMOS sensor, which is present on diagonals of

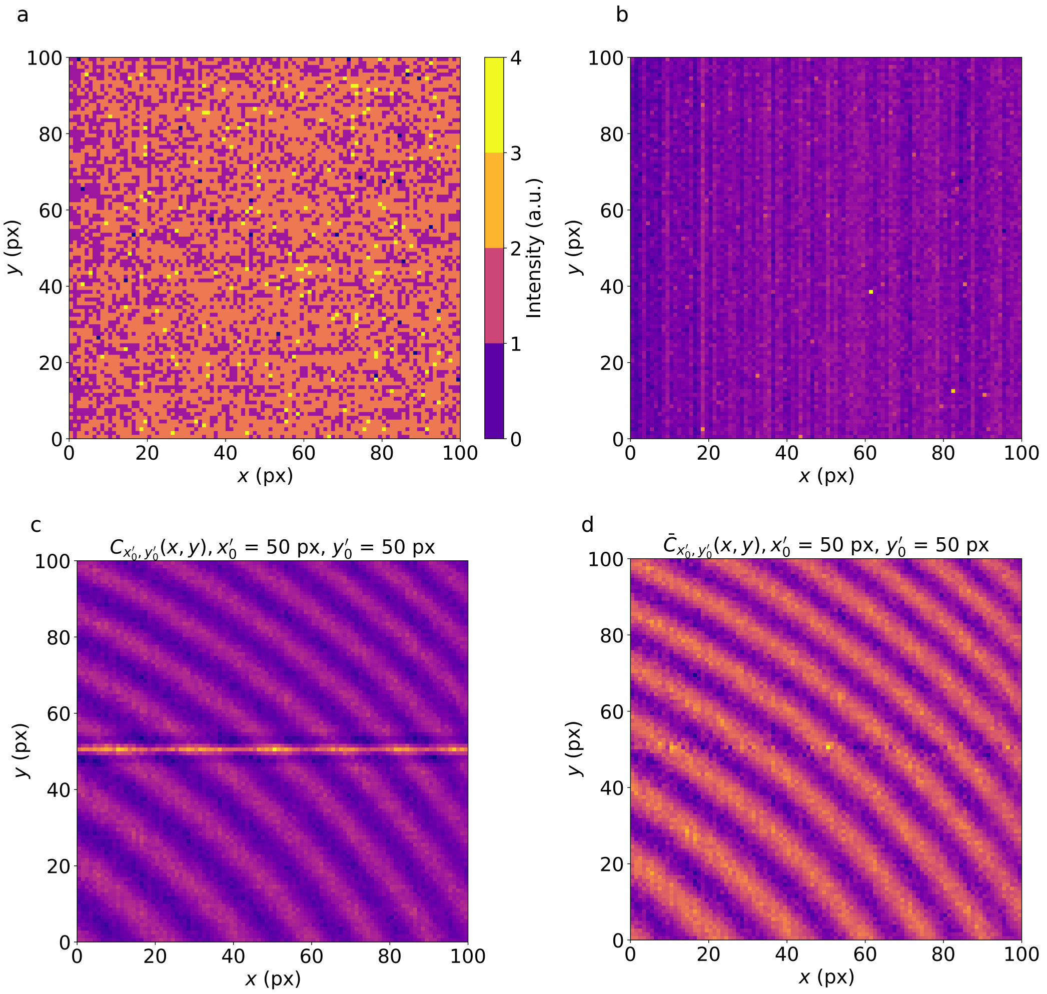

Figure 12: Data representation of noisy data measurement for 2D phase imaging. **(a)** presents a random noisy frame from the experiment; no fringes are visible on any of the recorded frames. **(b)** presents an average of over 135,854 recorded frames. **(c–d)** are the unnormalized ($C_{x0',y0'}(x,y)$) and normalized ($\bar{C}_{x0',y0'}(x,y)$) 2D cross-section of correlation maps through (x,y) subspace.

the 1D phase cross-sections – we replace diagonals with an average of the two neighboring pixels. We present the unnormalized and the normalized, corrected diagonals correlation map slices through (x, y) sub-space in Fig.12(c) and (d) respectively. For the clarity of the data analysis, we decided to omit this step, as this correction did not influence the phase retrieval process. For the phase retrieval, we proceed with a normalized 4D correlation map, with just diagonals corrected, presented through the slice in Fig.12(d).

Fourier analysis of the correlation map follows the exact steps described in S5.3. We

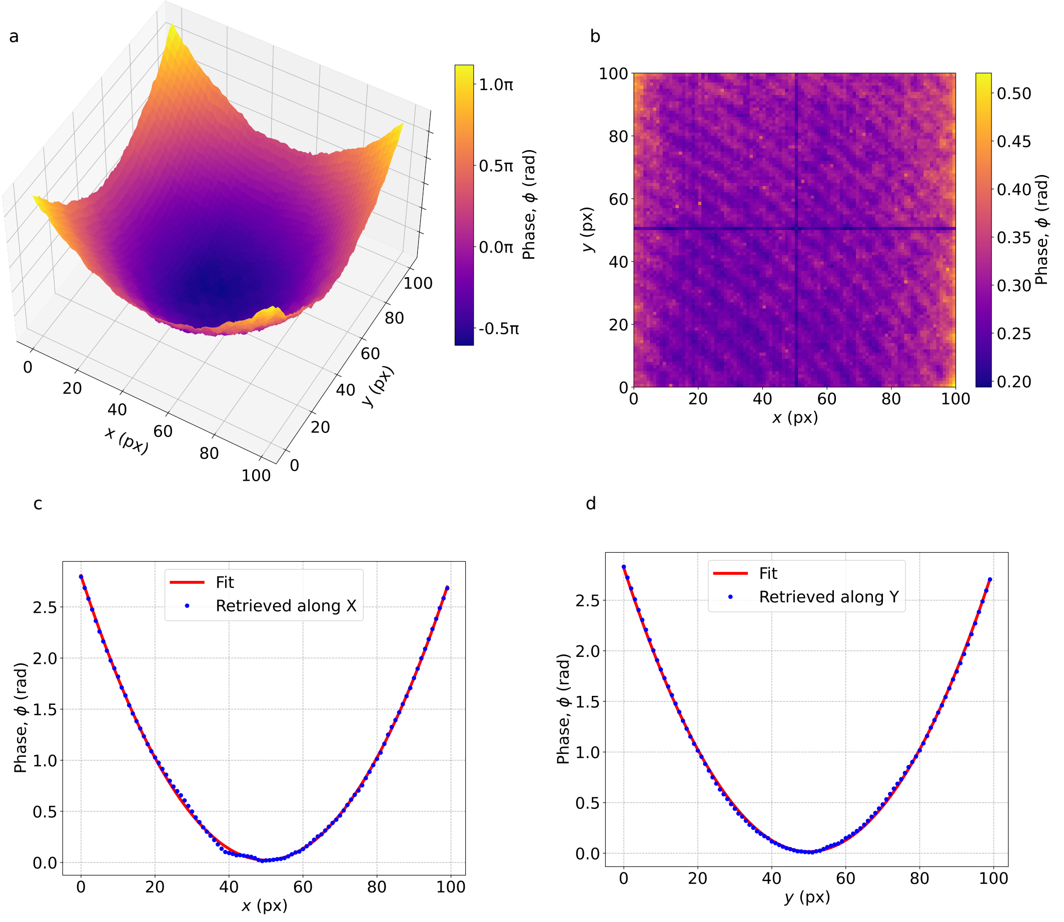

Figure 13: Reconstruction of noise resistant 2D phase measurement. **(a)** represents the reconstructed 2D quadratic phase. **(b)** is the standard deviation of the measured phase. **(c–d)** show the 1D averages of the measured phase fitted with quadratic function for both along the x and y-axis.

show the retrieved 2D phase with the linear phase removed in Fig.13(a), where Fig.13(b) is the calculated standard deviation of measured phase slices, and Fig.13(c - d) are the 1D averages of the measured phase with fitted quadratic function for x and y axis respectively. We measured the average standard deviation per pixel to be equal to 0.29 Radians. If we neglect systematic effect and assume that only statistics contributes to the uncertainty, this suggests that the average signal (averaged over almost 10 000 phase slices) will be around $\sqrt{N_{slices}} = \sqrt{10000} = 100$ times smaller, reaching about 0.003 Radians error per pixel. (Scaling calculation assumes a flat illumination profile, which was valid for this measurement).

S5.6 Simplified Correlation Map

Analysis of the 4D matrix requires a quadratic amount of resources and can be demanding for large matrices. We propose here a lighter approach to data analysis, where instead of creating an entire correlation map in 4D, we create only a single slice. We first gather the same frames as for the previously described method, the difference is only in the data analysis part.

We average all measured frames creating $\bar{I}(x, y)$ and find (x_{max}, y_{max}) – the point of highest intensity:

$$\bar{I}(x, y) = \frac{1}{N_{frames}} \sum_t \tilde{I}(x, y, t), \tag{46}$$

where $\tilde{I}(x, y, t)$ are frames measured at different moments of time t and N_{frames} is the number of frames. We then create a single 2D correlation map slice $C(x, y, x_{max}, y_{max})$:

$$C(x, y, x_{max}, y_{max}) = \frac{1}{N_{frames}} \sum_t \tilde{I}(x, y, t)\tilde{I}(x_{max}, y_{max}, t). \tag{47}$$

We choose a slice corresponding to x_{max}, y_{max} as the highest signal magnitude corresponds to the smallest error. The correlation matrix $C(x, y, x_{max}, y_{max})$ will contain a beam (and for the CMOS camera case the readout nonlinearities) and can be easily normalized using calculated $\bar{I}(x, y)$:

$$\bar{C}(x, y, x_{max}, y_{max}) = \frac{C(x, y, x_{max}, y_{max})}{\bar{I}(x, y)}. \tag{48}$$

The resulting matrix $\bar{C}(x, y, x_{max}, y_{max})$ is equal to the slice through the full 4D correlation map and can be analyzed through the standard off-axis holography method as a single phase measurement. It is worth noting, that in this case, we get a significantly lower amount of data, and therefore the measured phase standard deviation will be up to N times worse than for the full 4D correlation map analysis.

S5.7 2D Phase Analysis – Conclusions

We showed an algorithm for the creation and analysis of the 4D correlation maps. We demonstrated that phase retrieval can use all the data from the correlation map and that we benefit from including all the 2D slices of the 4D correlation map in the analysis. We successfully verified the algorithm both on perfect and noisy simulated data.

Finally, we demonstrated the measurement of a very weak signal, buried in the camera noise, and showed fringes visible in correlations as well as the retrieved phase. We also experimentally demonstrated that the proposed method of phase measurement can be implemented using a standard CMOS camera, therefore dramatically increasing the applicability of our method to solving real-life problems.
